# Antimicrobial resistance landscape and COVID-19 impact in Egypt, Iraq, Jordan, and Lebanon: A survey-based study and expert opinion

**DOI:** 10.1371/journal.pone.0288550

**Published:** 2023-07-27

**Authors:** Abdul Rahman Bizri, Alia Abd El-Fattah, Hafez Mahmoud Bazaraa, Jamal Wadi Al Ramahi, Madonna Matar, Rana Abdulmahdi Nahi Ali, Rowan El Masry, Jihane Moussa, Ali Jamal Al Abbas, Mohamed Abdel Aziz

**Affiliations:** 1 National COVID-19 Vaccine Committee, American University of Beirut Medical Center, Beirut, Lebanon; 2 Critical Care Department, Cairo University, Giza, Egypt; 3 Pediatric Critical Care Units, Department of Pediatrics, Faculty of Medicine, Cairo University, Giza, Egypt; 4 School of Medicine, University of Jordan, Amman, Jordan; 5 Notre Dame des Secours University Hospital, Byblos, Lebanon; 6 Holy Spirit University of Kaslik, Byblos, Lebanon; 7 Public Health Directorate, Ministry of Health, Baghdad, Iraq; 8 Medical Affairs, Pfizer, Egypt; 9 Pfizer Inc, Medical Affairs, Lebanon; 10 Medical Affairs, Pfizer, Iraq; 11 Pfizer Inc, Medical Affairs, United Arab Emirates; Zhejiang University, CHINA

## Abstract

**Objectives:**

The objective of this study was to assess the antimicrobial resistance (AMR) landscape and the impact of COVID-19 on AMR in Egypt, Iraq, Jordan, and Lebanon, and to gather expert opinions on the barriers to the implementation of antimicrobial stewardship (AMS) initiatives in the region.

**Methods:**

A cross-sectional questionnaire survey was used to assess the current AMR landscape, existing AMS initiatives, barriers to implementing AMS initiatives, and the impact of COVID-19 on AMR in the four countries.

**Results:**

The survey was completed by 204 physicians from Egypt (n = 82), Lebanon (n = 49), Iraq (n = 43), and Jordan (n = 30). Previous antibiotic use and previous bacterial colonization were perceived as the most common risk factors for an increase in AMR. According to the survey, multidrug-resistant (MDR) gram-negative bacteria were most common in lower respiratory tract infections, and *Klebsiella pneumoniae* and *Escherichia coli* were the most commonly identified gram-negative bacteria in hospital-acquired infections. Only 14.8% of pediatric physicians and 28.6% of adult physicians reported that target pathogen genotyping and phenotyping were done in hospitals, and the most commonly reported reasons for the lack of testing were technological and resource constraints. These constraints, coupled with the scarcity and high cost of newer antibiotics, have been identified as the most significant barriers to the successful management of MDR gram-negative bacterial infections in the region. It was reported that the spectrum of activity and safety of the antibiotic, the site of infection, the presence of comorbidities, and published guidelines and local antibiograms determined the choice of empirical antibiotic therapy for patients in the region. The four countries experienced a significant rise in AMR due to several factors during the COVID-19 pandemic, including an increase in hospital occupancy, a shift in priorities away from AMR surveillance, and changes in AMR epidemiology. Additionally, the large volumes of unnecessary and unsubstantiated antibiotic prescriptions during the COVID-19 pandemic has led to subsequent antibiotic shortages and significant increases in AMR in the region. Physicians also noted that the majority of COVID-19 patients were already on antibiotics before visiting the healthcare facility. MDR gram-negative bacteria were found in the majority of COVID-19 patients admitted to the intensive care unit. Despite the fact that various AMS initiatives have been implemented, they are not standardized across the region. Some of the main barriers to AMS implementation in the region are a lack of adequately trained AMS staff, lack of AMS knowledge and training among healthcare professionals, financial constraints, and the lack of AMR surveillance systems.

**Conclusion:**

These survey results provide valuable insights into the existing AMR and AMS landscape in the region, as well as the barriers that impede efficient AMS and AMR management. Based on these findings, the authors developed a call to action that suggests ways for each country in the region to address these challenges.

## Introduction

Antimicrobial resistance (AMR) has been identified as a major worldwide public health concern [1–4]. The increase in AMR has been attributed to the unnecessary use of antimicrobials. Due to the lengthier turnaround time of traditional antimicrobial susceptibility testing (AST), broad-spectrum antibiotics are often prescribed based on the presenting signs and symptoms of patients rather than using AST-guided prescriptions of targeted narrow-spectrum antibiotics [5–7]. There is also a general lack of awareness of the evolving AMR landscape, infection management, and development in diagnostic testing procedures among some physicians [6]. Furthermore, unregulated sales of antimicrobial agents without a medical prescription, self-medication, sub-standard drug quality, and non-adherence to approved dosing regimens all contribute to the existing AMR burden [3, 7]. The use of antibiotics in livestock and crops results in selective pressure, which leads to an increase in resistant bacteria that can be passed on to humans through food and the environment [7].

As one of the top ten global public health threats, AMR has been linked to prolonged illnesses and length of stay at the hospital, and greater mortality rates, resulting in increased healthcare costs and a general decrease in productivity owing to illness [1, 2]. In 2019, the global burden of AMR infections was estimated at 4.95 million deaths of which 1.27 million deaths were directly attributed to drug resistance [8]. Of these, over 100,000 deaths were attributable to methicillin-resistant *Staphylococcus aureus*. Additionally, six other pathogen–drug combinations involving MDR (excluding extensively drug-resistant) tuberculosis, cephalosporin-resistant *Escherichia coli*, carbapenem-resistant *Acinetobacter baumannii*, fluoroquinolone-resistant *E*. *coli*, carbapenem-resistant *Klebsiella pneumoniae*, and cephalosporin-resistant *K*. *pneumoniae* were each associated with 50,000 to 100,000 resistant-attributable deaths. Multidrug-resistance (MDR) infections in pediatric patients is estimated to represent up to 30% of the total number of MDR cases in Europe [9]. In the Middle East, most newborns (90%) in the intensive care unit (ICU) with sepsis had MDR bacteria and 66% of neonatal sepsis and meningitis in Sub-Saharan Africa were caused by bacteria that were resistant to antibiotics. In parts of Southeast Asia, 83% of children harbored *E*. *coli* that was resistant to first-line antibiotics, and 20% of pediatric patients in the United States developed resistance to colistin that was used to treat existing MDR gram-negative bacterial infections. The increased risk of antibiotic resistance in bacterial isolates recovered from the urinary tract of children who had previously received antibiotic treatment could persist for up to 6 months [10].

A systematic review of literature evaluating the AMR landscape based on hospital surveillance in the Arab region, including Egypt, Iraq, Jordan, and Lebanon, between 2000 and 2020 provided insights into the AMR landscape in the region [11]. The systematic review identified an increase in reported bacterial resistance to cephalosporins (from 37% to 89.5%), fluoroquinolones (from 46.8% to 70.3%), aminoglycosides (from 40.2% to 64.4%), monobactams (from 30.6% to 73.6%), and carbapenems (from 30.5% to 64.4%) in the Arabian region between 2000 and 2020. Another systematic review of literature identified an increase in AMR of *Acinetobacter* species, *Enterobacteriaceae* (*E*. *coli* and *Klebsiella* species) and *Pseudomonas aeruginosa* to cephalosporins, carbapenems, and colistin in Iraq, Jordan, and Lebanon between 2015 and 2020 [12]. The most commonly observed AMR mechanisms in gram-negative bacteria were genetic modifications leading to an increased expression of antimicrobial-inactivating enzymes and decreased permeability.

The COVID-19 pandemic has dramatically changed the global healthcare landscape [13]. A meta-analysis of 154 studies that evaluated the proportion of COVID-19 patients who were prescribed an antibiotic, reported that 74.6% of 30,623 patients received antibiotics and prescriptions were higher among older patients and those with more severe COVID-19 symptoms. Reported bacterial co-infections were estimated between 6.1% and 8.0%. Therefore, most antibiotic prescriptions were unnecessary. The high volumes of unnecessary antibiotic use in COVID-19 infections can increase selective pressures on bacterial populations leading to the emergence of AMR and subsequent genetic transmission of AMR traits across different bacterial species [13, 14]. In the Middle East and North Africa, many countries lack the resources to implement an effective and reliable AMR surveillance system and to provide institutions with rapid and sensitive diagnostic tools [15, 16]. Due to the lack of antimicrobial stewardship (AMS) initiatives, empirical antibiotics are used inefficiently and redundantly rather than being prescribed based on microbial surveillance and resistance patterns [16]. By implementing evidence-based initiatives, the AMS program aims to promote the judicious use of antimicrobial drugs across healthcare [17–19]. AMS implementation strategies are a step-by-step dynamic process that is specifically tailored to the available resources and patient outcomes in the regional facilities [20, 21]. The three pillars to an integrated strategy to bolster healthcare systems includes AMS, infection prevention and control, and medicine and patient safety [20]. The Global Action Plan for AMR was developed by the World Health Organization (WHO) with a set of five strategic objectives: i) improving AMR awareness and understanding by means of effective communication, education, and training; ii) surveillance- and research-based evidence generation; iii) effective, sanitation, hygiene, and infection control; iv) optimal antibiotic use; v) develop sustainable financial strategies to fund newer medicines, diagnostic tools, vaccines, and other interventions.

By 2050, it is estimated that roughly 10 million people would have died annually from AMR [8]. The WHO created the Global Antimicrobial Resistance and Use Surveillance System in 2015, with the goal of standardizing AMR surveillance and evaluating AMR interventions worldwide [22]. By 2020, 127 countries were enrolled in the program, including Egypt, Iraq, Jordan, and Lebanon [23]. The region is home to a diverse population of people with varying cultural and ethnic backgrounds and displays notable differences in resources, development, and healthcare spending [24]. Furthermore, political and economic unrest has strained the healthcare system, leading to a significant lag in the development of AMR surveillance in comparison to developed nations. Antibiotic prescription practices are inconsistent and the sale of antibiotics is unregulated. The AMR landscape has significantly been impacted by the notable rise in antibiotic usage during the COVID-19 pandemic which could be attributed to the similarity in the clinical presentation of COVID-19 and bacterial respiratory infections. A review of published literature reported that of the 72% of hospitalized COVID-19 patients who received antimicrobial therapy, only 8% had documented bacterial and/or fungal coinfection. There is limited published data on the impact of COVID-19 on the AMR landscape in the Arab region which is estimated to be magnified compared to other developed regions.

This survey-based cross-sectional study aims to evaluate and comprehend the existing AMR landscape in the clinical setting in Egypt, Iraq, Jordan, and Lebanon in terms of the management of AMR gram-negative infections and AMS implementation based on insights obtained from the perspectives of physicians in the region. This study also aims to understand the impact of the COVID-19 pandemic on the existing AMR and AMS landscape in these countries.

## Methods

A working group comprising of members who were selected based on their expertise in the management of MDR infections from Egypt, Iraq, Jordan, and Lebanon was established. Each expert was either a leading infectious disease researcher or an eminent clinical microbiologist or intensivist from their respective country. An anonymous online survey was developed with the aim of gathering insights from physicians in the region about the local epidemiology and management strategies for multidrug-resistant (MDR) bacterial infections, as well as the AMS practices in Egypt, Iraq, Jordan, and Lebanon. The survey comprised of 30 multiple-choice questions (MCQs) tailored for pediatric physicians and 30 MCQs for adult physicians and included: 16 rank order scale questions, 4 Likert scale questions, 4 slider scale questions, 6 MCQs with the option to select a single response (S2 Appendix). Of the 30 questions, 14 questions allowed the selection of multiple responses and 16 questions allowed a single response to be selected. The questionnaire was specifically designed to gain insights into the perceptions and experiences of physicians practicing in the region about the current regional AMR landscape, diagnostic and management strategies for severe MDR gram-negative bacterial infections, AMS initiatives, and the impact of the COVID-19 pandemic on AMR epidemiology and management.

The survey was critically evaluated by the working group to ensure relevance, clarity, conciseness, and ease of completion and was piloted with 10 external participants to determine the scope and clarity of the questions, and to confirm its face validity. The questions were revised based on the responses and feedback from the pilot study. These responses were excluded from the final analyses. The questionnaire was programmed into SurveyMonkey®, and a link to the survey was circulated by email to physicians, including infectious disease specialists, internal medicine specialists, microbiologists and intensivists, pulmonologists, neonatologists, and pediatricians in Egypt, Iraq, Jordan, and Lebanon, who were identified through regional societal mailing lists. Following the initial invitation, two follow-up reminders were sent to the participants by email. The anonymity of the participants was maintained throughout the survey and the study. This survey was conducted from March 16, 2022, to June 10, 2022. Only completed survey responses were included in the study.

This anonymous survey-based, observational, non-interventional study was aimed at gathering insights regarding the regional AMR and AMS landscape and practices based on the personal subjective experiences and understanding of pediatric and adult physicians in the region without the need to refer or assess patient, hospital, or laboratory records. The objective of the study is to contribute to the healthcare knowledge base and to provide useful information to support healthcare providers in their clinical practice. The study was conducted in accordance with the Declaration of Helsinki and did not involve any patients. No interventions on human participants or animals were performed by any of the authors, and no personal information or records of the participating physicians or their patients were obtained or accessed during the study. All the participating physicians were informed of the purpose of the study, the methods used, and their personal right to decline or withdraw from the study at any time. The participating physicians who completed the survey provided their virtual consent by clicking on the ’I agree’ button in the information page at the start of the survey, which confirmed their agreement to the consent statement, their willingness to participate in the anonymous study survey, and the subsequent publication of the data. Only those physicians who provided their informed consent were subsequently directed to the online survey questionnaire. All collected information was kept confidential and anonymous to protect the privacy of the participants, and the data was analyzed anonymously. Therefore, in this context, approval by an institutional ethical committee was not obtained for the study [25].

### Statistical analysis

The survey included MCQs that allowed participants to select more than one option that was applicable to each question. The survey also included questions that required participants to rank or categorize the provided options. In order to improve the accuracy of the survey data, analysis included descriptive statistics, frequency proportions, and median interquartile statistics. All statistical analyses were performed using StataIC (StataCorp, Version 16).

## Results

### Survey participants

The survey was sent to 547 physicians from Egypt, Iraq, Jordan, and Lebanon, who were identified through regional societal mailing lists (S1 and S2 Tables in S1 Appendix). A total of 204 (37.2%) physicians (Egypt, n = 82; Lebanon, n = 49; Iraq, n = 43; and Jordan, n = 30) completed the survey (S1 and S2 Tables in S1 Appendix). Of these, 50 (24.5%) were pediatric physicians and 154 (75.5%) were adult physicians (S2 Table in S1 Appendix). They included intensivists (23%), infectious disease specialists (19.6%), and chest physicians/pulmonologists (11.7%), as well as other specialties (S2 Table in S1 Appendix). Incomplete survey responses were excluded from the study.

### Epidemiology of AMR

#### Risk factors for MDR and carbapenem-resistant gram-negative bacterial infections

*What are the most common risk factors for MDR gram-negative bacterial infections in your hospital*? Physician responses: pediatric, 48 of 50 (96%); adult, 152 of 154 (98.7%).

The most commonly perceived risk factors for MDR gram-negative bacterial infections reported by the participating physicians in the four countries were prior infection or colonization with MDR gram-negative bacteria, or prolonged hospitalization, as well as treatment with antibiotics within the last 90 days (S3 Table in S1 Appendix).

*What are the most common risk factors for carbapenem-resistant gram-negative bacterial infections in your hospital*? Physician responses: pediatric, 49 (98%); adult, 145 (94.1%).

The use of carbapenem or other antibiotics in the past, previous carbapenem-resistant gram-negative bacterial colonization, previous ICU hospitalization, and prolonged stay at the hospital were perceived by physicians as the most common risk factor for carbapenem-resistant gram-negative bacterial infections among patients. Mechanical ventilation and antibiotic treatment within the last 90 days were also perceived to be associated with an increased risk of infections among patients in the region (S3 Table in S1 Appendix).

#### Prevalence of MDR gram-negative bacteria

*In your hospital*, *MDR bacteria are most frequently encountered in which type of infections*? Physician responses: pediatric, 50 (100%); adult, 154 (100%).

**Fig 1:**
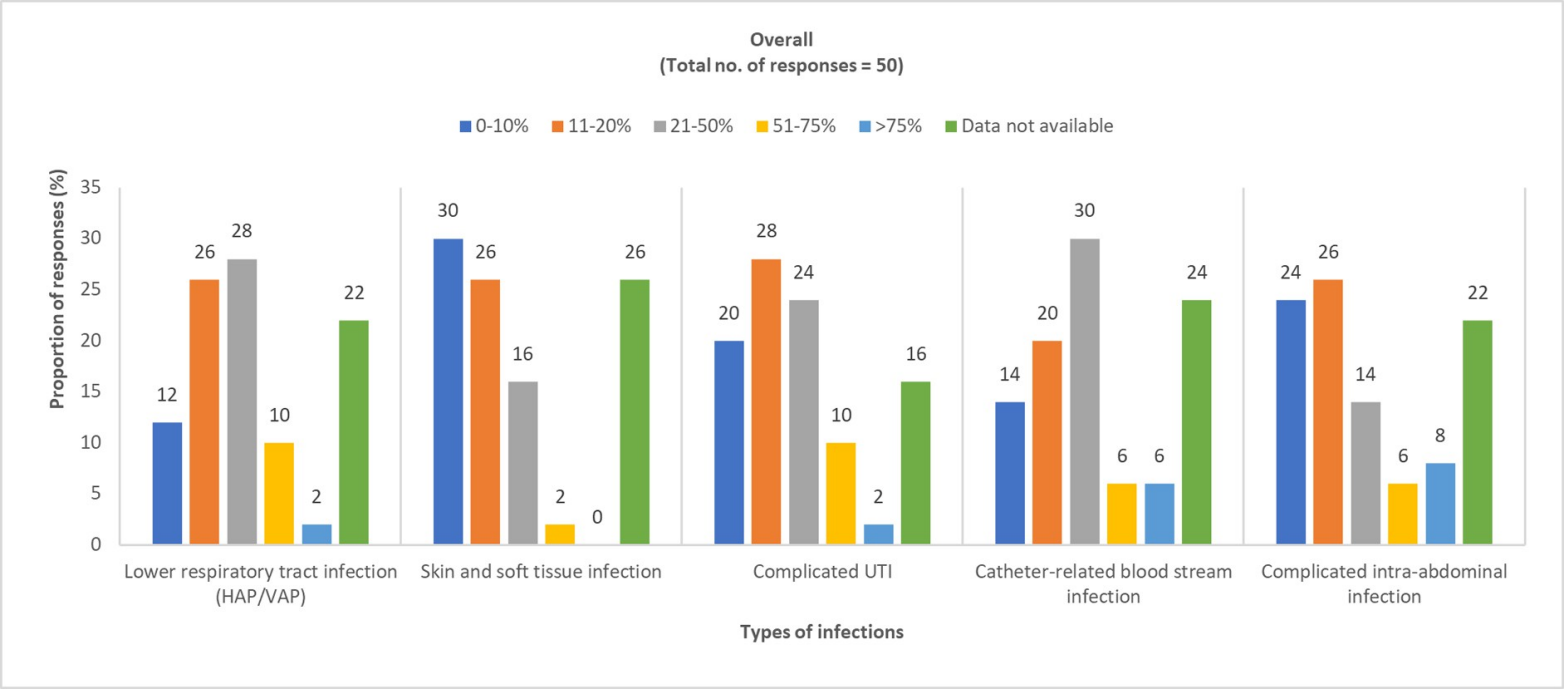
Physician-perceived prevalence of MDR gram-negative bacteria among pediatric patients. HAP, hospital-acquired pneumonia; MDR, multidrug resistant; UTI, urinary tract infection; VAP, ventilator-associated pneumonia.

**Fig 2:**
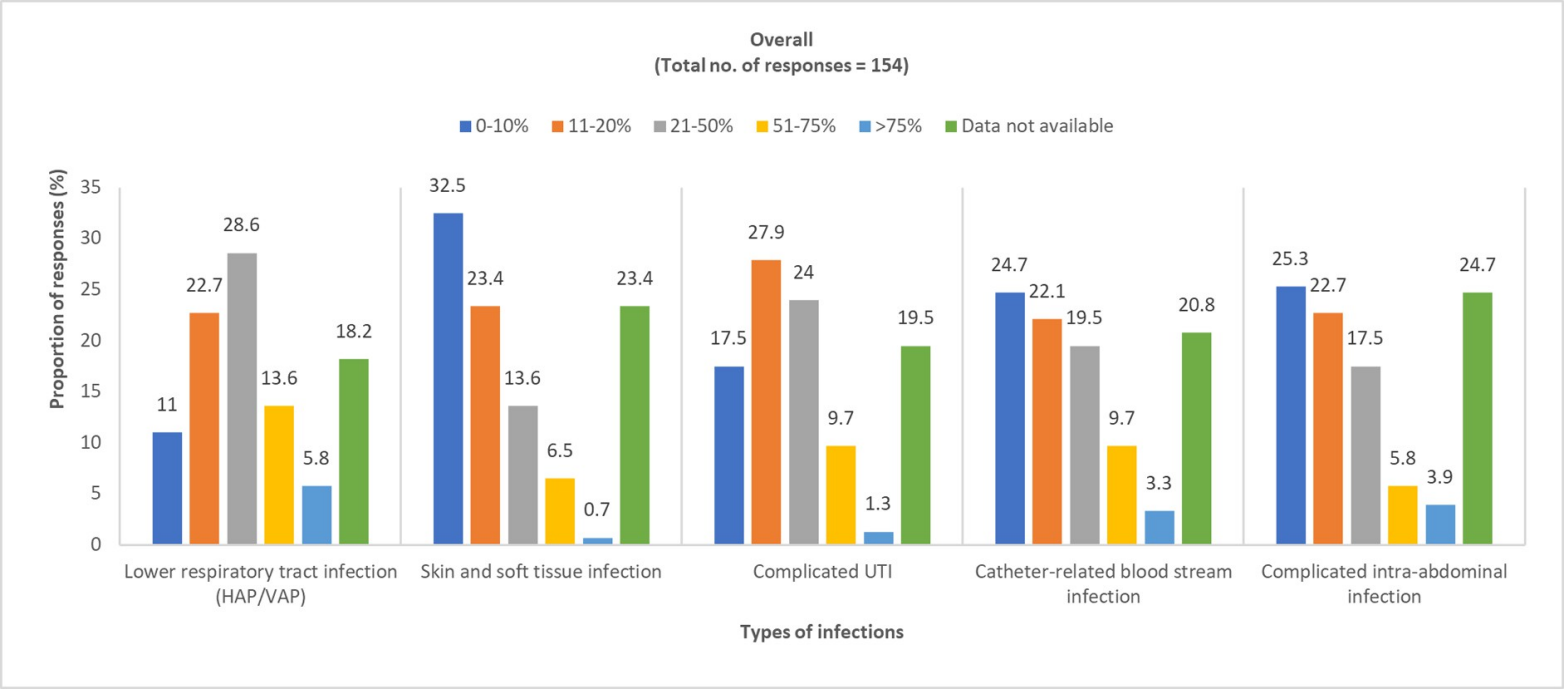
Physician-perceived prevalence of MDR gram-negative bacteria among adult patients. HAP, hospital-acquired pneumonia; MDR, multidrug resistant; UTI, urinary tract infection; VAP, ventilator-associated pneumonia.

Overall, the perception of the physicians on the prevalence of MDR gram-negative bacteria in different types of infections varied across the region as shown in Figs 1 and 2 (S4 Table and S1 Fig in S1 Appendix).

#### Prevalent hospital-acquired MDR gram-negative bacteria and AMR mechanisms

*Of all the HAIs in your patients*, *which gram-negative bacteria are most commonly identified*? Physician responses: pediatric, 50 (100%); adult, 146 (94.8%).

Overall, in Egypt, Iraq, Jordan, and Lebanon, the physicians perceived *K*. *pneumoniae*, *P*. *aeruginosa*, *E*. *coli*, and *A*. *baumannii* to be the most prevalent gram-negative bacteria in HAIs. However, the physicians’ estimations of the proportion of prevalence varied significantly by country (S6 Table in S1 Appendix).

*What is the prevalence of AMR mechanisms in hospital acquired gram-negative bacterial infections*? This question had multiple parts and the number of respondents for each was different. The number of pediatric and adult respondents for each part of the question is provided in S5 Table in S1 Appendix.

**Fig 3:**
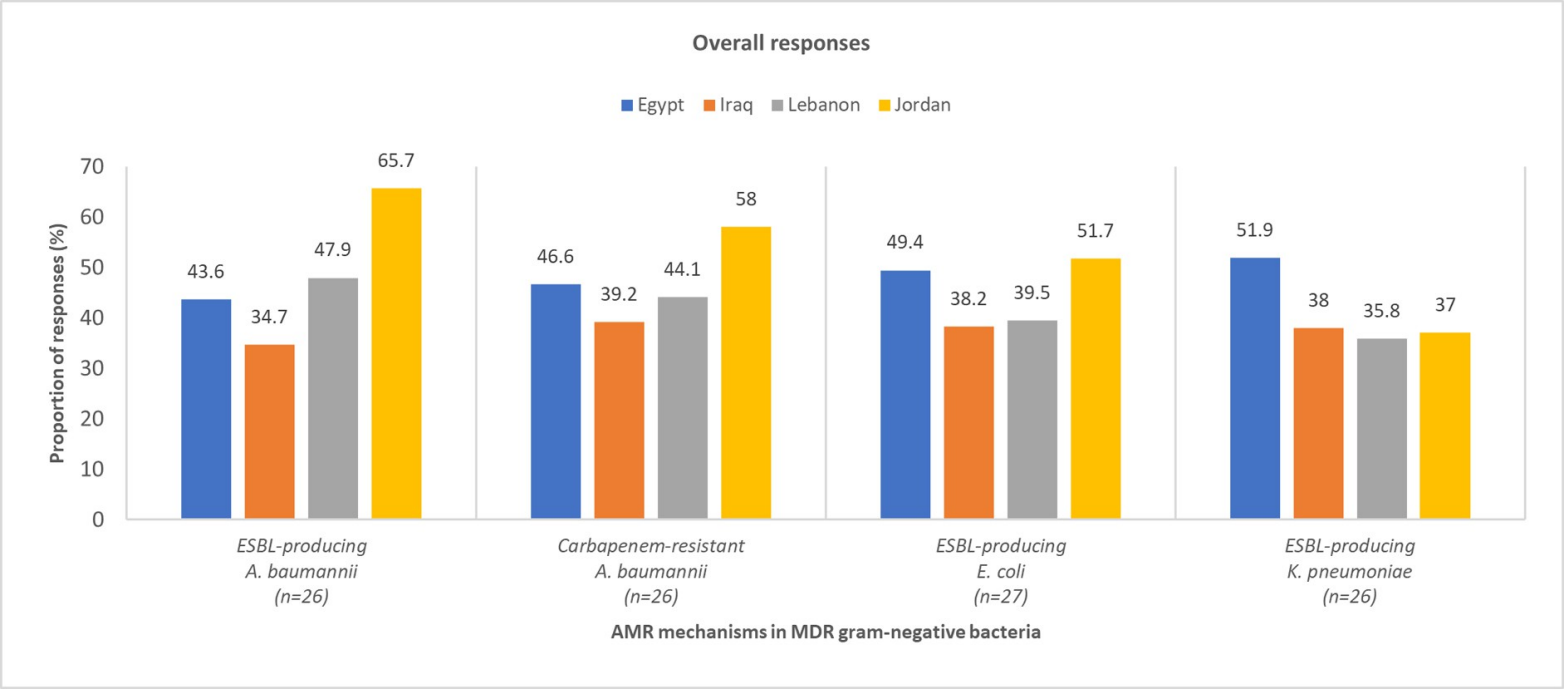
Physician-perceived prevalence of AMR mechanisms in pediatric HA MDR gram-negative bacterial infections. *A*. *baumannii*, *Acinetobacter baumannii*; AMR, antimicrobial resistance; *E*. *coli*, *Escherichia coli*; ESBL, extended-spectrum β-lactamase; HA, hospital-acquired; *K*. *pneumoniae*, *Klebsiella pneumoniae*; MDR, multidrug-resistant.

**Fig 4:**
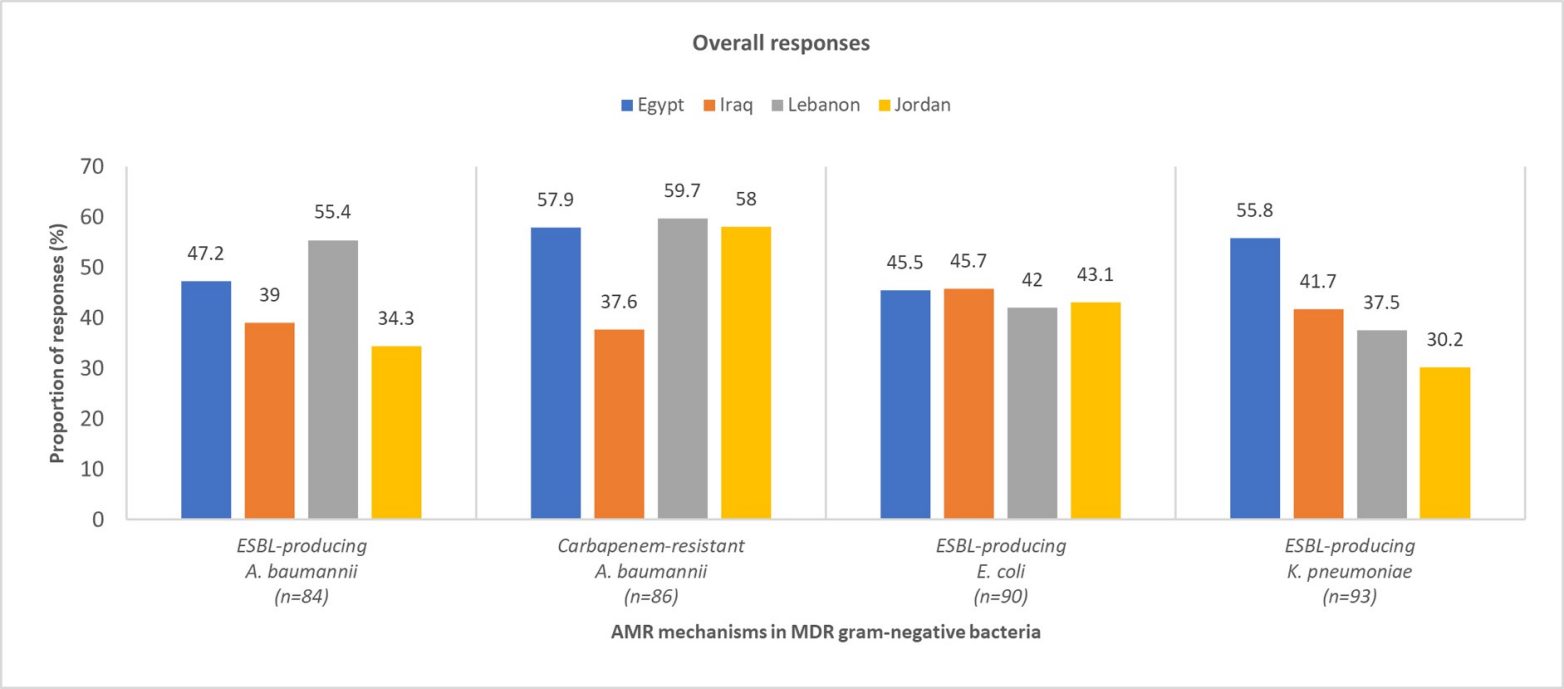
Physician-perceived prevalence of AMR mechanisms in adult HA MDR gram-negative bacterial infections. *A*. *baumannii*, *Acinetobacter baumannii*; AMR, antimicrobial resistance; *E*. *coli*, *Escherichia coli;* ESBL, extended-spectrum β-lactamase; HA, hospital-acquired; *K*. *pneumoniae*, *Klebsiella pneumoniae*; MDR, multidrug-resistant.

Overall, most physicians from the region were of the opinion that extended-spectrum β-lactamase (ESBL) production and carbapenem-resistance were the most prevalent AMR mechanisms among *A*. *baumannii*, *E*. *coli*, and *K*. *pneumoniae* in HAIs among pediatric (Fig 3) and adult (Fig 4) patients (S5 Table in S1 Appendix). According to the survey, there were large variations in the physicians’ estimations of the most prevalent AMR mechanisms among gram-negative bacterial HAIs. This could be attributed to the poor AMR surveillance and scarcity of available data in the region.

*In your hospital*, *what is the prevalence of enzyme coding gene mutations leading to AMR in MDR gram-negative bacteria*? Physician responses: pediatric, 50 (100%); adult, 154 (100%).

**Fig 5:**
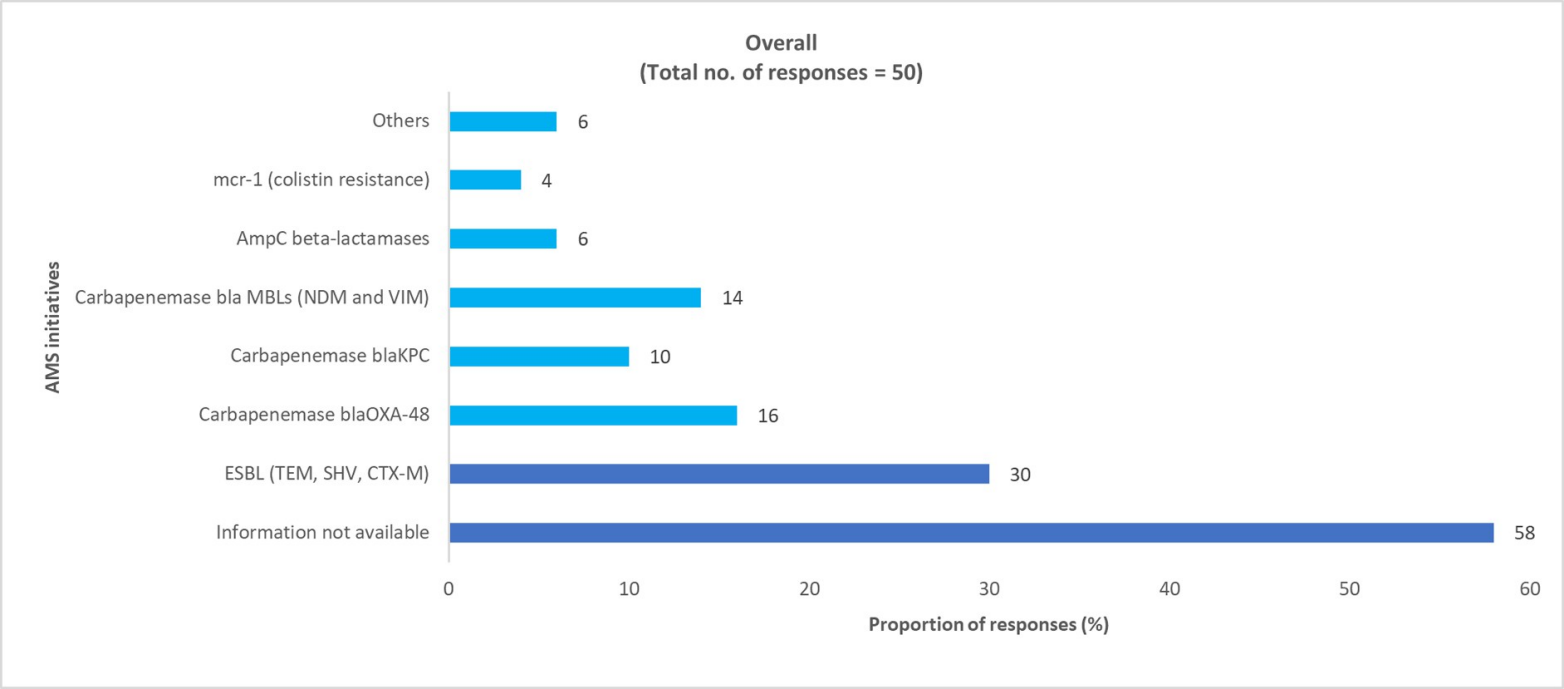
Physician-perceived prevalence of AMR enzyme-coding gene mutations in pediatric HA MDR gram-negative bacteria. AMR, antimicrobial resistance; ESBL, extended-spectrum β-lactamase; HA, hospital-acquired; MDR, multidrug-resistant.

**Fig 6:**
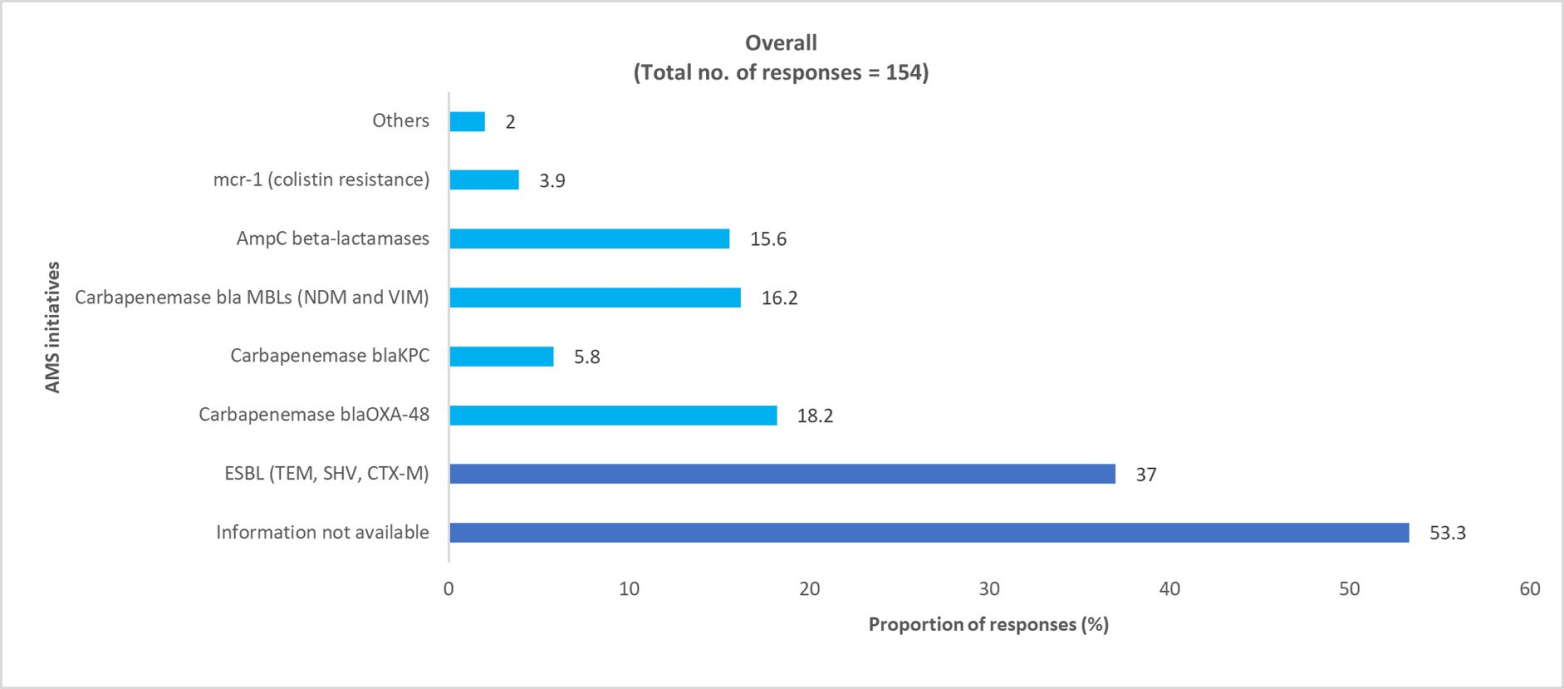
Physician-perceived prevalence of AMR enzyme-coding gene mutations in adult HA MDR gram-negative bacteria. AMR, antimicrobial resistance; ESBL, extended-spectrum β-lactamase; HA, hospital-acquired; MDR, multidrug-resistant.

Overall, 58% of pediatric physicians and 53% of adult physicians reported that data regarding the prevalence of AMR enzyme-coding gene mutations was not available (S6 Table and S2 Fig in S1 Appendix). In the survey, the physicians noted that MDR gram-negative bacteria with ESBL and carbapenemase enzyme-coding gene mutations were most commonly encountered in clinical practice (Figs 5 and 6 and S6 Table in S1 Appendix).

#### AMR diagnosis and testing

*Does the laboratory in your hospital conduct both genotypic and phenotypic testing of target microorganisms*? Physician responses: pediatric, 47 (94%); adult, 143 (92.8%).

The survey identified that genotyping and phenotyping practices were not standardized across the region, and according to most of the physicians (pediatric, 72.3%; adult, 51.7%), genotyping and phenotyping was not done in their respective hospitals (S7 Table in S1 Appendix). Surprisingly, 12.7% and 19.5% of pediatric and adult physicians, respectively, reported that they were unaware about the availability of genotyping and phenotyping facilities in their respective institutions.

#### Barriers to routine antimicrobial susceptibility testing (AST)

*What are the barriers to ideal AST at your institution/hospital*? Physician responses: pediatric, 45 (90%); adult, 133 (86.3%).

According to the survey, most physicians in Egypt and Iraq were of the opinion that AST technological limitations were the biggest barrier to routine AST in pediatric practice, while high costs associated with microbiological diagnosis and AST and lack of access to well-equipped microbiological laboratories were the biggest barriers in Jordan and Lebanon, respectively (S8 Table in S1 Appendix). In adult practice, limitations in AST technology and the unavailability of advanced diagnostic equipment in microbiological laboratories were perceived as the most significant barriers to routine AST. Additionally, the survey identified the high costs associated with AST as a major limiting factor in Egypt, Jordan, and Lebanon. These impediments, combined with the absence of robust communication policies and insufficient sampling and reporting, are likely associated with lower frequencies of AST in the region.

*When do you generally order microbial culture and AST of bacterial pathogens within your hospital/center*? Physician responses: pediatric, 46 (92%); adult, 138 (89.6%).

Most physicians in the region reported that they ordered microbial culture and AST for all pediatric patients requiring antibiotic treatment, while some physicians reserved testing only for infected ICU patients and those who were at a high risk of MDR infection (S8 Table in S1 Appendix). According to the survey, most physicians in Egypt noted that they ordered microbial culture and AST for all adult patients in the ICU with suspected infection despite empirical antibiotic therapy (S8 Table in S1 Appendix). In Jordan and Lebanon, most physicians stated that the choice of antibiotic treatment in adult patients, including infected ICU patients, was informed by prior microbial culture and AST testing. In Iraq, only patients who failed initial antibiotic therapy were tested. The survey identified that microbial culture and AST practices varied significantly in adult clinical practice across the region.

Based on the survey, it was noted that significant barriers hindered ideal AST practices across the region. Many physicians noted that they ordered microbial culture and AST for specific types of patients, particularly if they are at an increased risk of MDR gram-negative bacterial infections.

### Medical management

#### Barriers to successful medical management of MDR gram-negative bacterial infections

*What are the barriers to the successful treatment or medical management of MDR gram-negative bacterial infections*? Physician responses: pediatric, 46 (92%); adult, 135 (87.6%).

The physicians ranked the lack of access to well-equipped laboratories and advanced microbiological diagnostic technologies (pediatric, 6.1%; adult, 5.5%), as well as the unavailability and high cost of newer antibiotics (pediatric 5.4%; adult, 5.6%) to be the most significant barriers to successful management of MDR gram-negative bacterial infections in Egypt, Iraq, Jordan, and Lebanon (S9 Table in S1 Appendix). Other reported barriers include a lack of awareness of the prevalence of MDR gram-negative bacteria, the scarcity of infectious disease specialists, and the absence of infection management guidelines/policies at both regional and national levels.

#### Considerations for empirical antibiotic therapy

*Which factors do you consider when choosing the appropriate empirical antibiotic therapy*? Physician responses: pediatric, 46 (92%); adult: 131 (85%).

According to the survey, physicians reported often using the spectrum and safety of the antibiotic, site of infection, presence of comorbidities, and published guidelines and local antibiograms to inform the type of empirical antibiotic prescription in the region (S9 Table in S1 Appendix). The history of exposure to antibiotics over the past 90 days as well as the pharmacology of the drug were also taken into consideration.

#### Treatment of severe MDR gram-negative bacterial infections

*What is your preferred treatment option for severe infections caused by ESBL-producing* Enterobacteriaceae (E. coli, K. pneumoniae, Enterobacter, *etc*.*)*, *carbapenem-resistant* Enterobacteriaceae, *MDR* P. aeruginosa? Physician responses: pediatric, 47 (94%); adult, 143 (92.8%).

Carbapenems were the preferred first-line treatment option for ESBL-producing *Enterobacteriaceae* infections according to the participants (Tables 1 and 2; S10-S12 Tables in S1 Appendix). Though new and conventional β-lactam/β-lactamase inhibitors (ceftazidime-avibactam and ceftolozane-tazobactam) and aminoglycosides were also used in the first-line setting, some physicians reserved these for second-line treatment. Severe carbapenem-resistant *Enterobacteriaceae* infections were commonly treated with new β-lactam/β-lactamase inhibitor, aminoglycoside monotherapy or its combinations, and polymyxin combination therapy; however, these were sometimes used in second-line and third-line settings as well (Tables 1 and 2; S10-S12 Tables in S1 Appendix). Tigecycline and polymyxin were also used in patients who were unresponsive to first-line treatment.

**Table 1:** Preferred choice of antibiotics for the treatment of pediatric MDR gram-negative bacterial infections by country as reported by the survey participants.

Antimicrobial treatment	Antimicrobial treatment by country											
	Egypt			Iraq			Jordan			Lebanon		
	First-line	Second-line	Third-line	First-line	Second-line	Third-line	First-line	Second-line	Third-line	First-line	Second-line	Third-line
Number of responses	22			12			6			7		
**Severe ESBL-producing *Enterobacteriaceae* infections**												
New BL-BLI^a^ (%)	18.1	22.7	NA	8.3	16.6	NA	33.3	16.6	NA	0	14.2	NA
Conventional BL-BLI^b^ (%)	22.7	9		16.6	0		0	16.6		57.1	0	
Aminoglycoside (%)	0	18.1		50	16.6		0	16.6		0	28.5	
Carbapenem (%)	59	22.7		25	33.3		50	16.6		42.8	28.5	
Aminoglycoside combinations (%)	NA	13.6		NA	25.00		NA	33.33		NA	28.5	
**Severe carbapenem-resistant *Enterobacteriaceae* infections**												
New BL-BLI^a^ (%)	22.7	18.1	18.1	25	41.6	25	33.3	0	0	14.2	14.2	28.5
Polymyxin (%)	22.7	9	13.6	0	8.3	0	0	0	0	0	0	0
Aminoglycoside combinations (%)	27.2	22.7	27.2	33.3	25	16.6	16.6	50	16.6	0	57.1	14.2
Polymyxin combinations (%)	13.6	22.7	9	0	0	0	50	16.6	33.3	14.2	0	0
**Severe MDR *Pseudomonas aeruginosa* infections**												
Carbapenem (%)	18.1	0	0	16.6	41.6	33.3	0	16.6	0	28.5	14.2	0
Other classical anti-pseudomonal β-lactam antibiotics (%)	27.2	9	0	25	25	25	0	16.6	16.6	14.2	42.8	0
Ceftazidime-avibactam (%)	9	22.7	18.1	8.3	0	0	33.3	16.6	16.6	0	14.2	28.5
Colistin with carbapenem/aminoglycoside combination (%)	31.8	40.9	31.8	8.3	8.3	8.3	0	16.6	16.6	28.5	0	0
Aminoglycoside with carbapenem combination (%)	9	13.6	4.5	16.6	8.3	0	16.6	33.3	33.3	0	14.2	0

BL-BLI, β-lactam/β-lactamase inhibitor; ESBL, extended-spectrum β-lactamase; MDR, multidrug-resistant.

^a^New BL-BLI include ceftazidime-avibactam and ceftolozane-tazobactam

^b^Examples of conventional BL-BLI include piperacillin-tazobactam, amoxicillin-clavulanic acid, and ampicillin-sulbactam. Note: ‘Not applicable’ implies that a drug(s) or combination(s) was not considered in a particular line of treatment.

**Table 2:** Preferred choice of antibiotics for the treatment of adult MDR gram-negative bacterial infections by country as reported by the survey participants.

Antimicrobial treatment	Antimicrobial treatment by country											
	Egypt			Iraq			Jordan			Lebanon		
	First-line	Second-line	Third-line	First-line	Second-line	Third-line	First-line	Second-line	Third-line	First-line	Second-line	Third-line
Number of responses	53			30			21			39		
**Severe ESBL-producing *Enterobacteriaceae* infections**												
New BL-BLI^a^ (%)	26.4	22.6	NA	46.6	23.3	NA	19	28.5	NA	33.3	46.1	NA
Conventional BL-BLI^b^ (%)	18.8	1.8		26.6	10		33.3	14.2		12.8	5.1	
Aminoglycoside (%)	0	11.3		0	3.3		0	0		2.5	5.1	
Carbapenem (%)	45.4	28.3		23.33	16.6		42.8	33.3		48.7	38.4	
Aminoglycoside combinations (%)	NA	18.8		NA	26.6		NA	9.5		NA	2.5	
**Severe carbapenem-resistant *Enterobacteriaceae* infections**												
New BL-BLI^a^ (%)	37.7	20.7	20.7	26.6	13.3	13.3	47.6	28.5	28.5	71.7	30.7	28.2
Aminoglycoside (%)	1.8	3.7	3.7	10	20	0	9.5	14.2	9.5	0	2.5	2.5
Polymyxin (%)	5.6	11.3	11.3	0	0	3.33	0	23.8	9.5	5.1	10.2	7.6
Tigecycline (%)	13.2	11.3	11.3	20	20	26.6	9.5	14.2	4.7	5.1	15.3	10.2
Aminoglycoside combination (%)	9.4	18.8	18.8	20	23.3	16.6	4.7	9.5	14.2	5.1	15.3	28.2
Polymyxin combination (%)	24.5	22.6	22.6	0	0	10	23.8	9.5	14.2	10.2	20.1	12.8
**Severe MDR *Pseudomonas aeruginosa* infections**												
Polymyxin (%)	9.4	7.5	9.4	3.3	3.3	3.3	28.5	23.8	19	7.6	5.1	15.3
Carbapenem (%)	20.7	3.7	0	33.3	13.3	10	4.7	9.5	0	7.6	5.1	5.1
Other classical anti-pseudomonal β-lactam antibiotics^c^ (%)	16.9	7.5	9.4	23.3	30	16.6	23.8	14.2	14.2	17.9	12.8	12.8
Ceftazidime-avibactam (%)	13.2	16.9	24.5	10	6.6	6.6	9.5	23.8	9.5	25.6	28.2	20.5
Ceftolozane-tazobactam (%)	1.8	7.5	3.7	3.3	3.3	10	4.7	0	4.7	23	12.8	10.2
Colistin with carbapenem/aminoglycoside combination (%)	24.5	30.1	18.8	10	13.3	16.6	19	19	28.5	10.2	23	15.3
Aminoglycoside with carbapenem combination (%)	9.4	18.8	13.2	10	20	13.3	4.7	0	14.2	5.1	7.6	5.1

BL-BLI, β-lactam/β-lactamase inhibitor; ESBL, extended-spectrum β-lactamase; MDR, multidrug-resistant; NA, not applicable.

^a^New BL-BLI include ceftazidime-avibactam and ceftolozane-tazobactam

^b^Examples of conventional BL-BLI include piperacillin-tazobactam, amoxicillin-clavulanic acid, and ampicillin-sulbactam

^c^Other classical anti-pseudomonal β-lactam antibiotics include aztreonam, cefepime, ceftazidime, and piperacillin-tazobactam. Note: ‘Not applicable’ implies that a drug(s) or combination(s) was not considered in a particular line of treatment.

Carbapenems, other classical anti-pseudomonal β-lactam antibiotics (aztreonam, cefepime, ceftazidime, and piperacillin-tazobactam), combinations of colistin and carbapenem, carbapenem/aminoglycoside, ceftazidime-avibactam, or monotherapy with polymyxin were used in the first-line treatment of severe MDR *P*. *aeruginosa* infections (Tables 1 and 2; S10-S12 Tables in S1 Appendix). Combinations of Fosfomycin with carbapenem/β-lactam antibiotics/aminoglycosides/colistin, colistin with carbapenem/aminoglycoside, or aminoglycoside with carbapenem were commonly used in the third-line setting (Tables 1 and 2; S10-S12 Tables in S1 Appendix).

### Impact of COVID-19 on the AMR landscape

#### Has the COVID-19 pandemic impacted AMR and the treatment of MDR gram-negative bacterial infections?

Physician responses: pediatric, 47 (94%); adult, 140 (90.9%).

Based on the survey, 38.3% of pediatric physicians and 68.5% of adult physicians were of the view that the COVID-19 pandemic had impacted the regional AMR landscape, bacterial epidemiology, and the management of MDR bacterial infections, where patients with active or prior COVID-19 infections had a particularly higher incidence of AMR (S13 Table in S1 Appendix). 51% and 21.4% of pediatric and adult physicians reported unavailability of regional data on the impact of COVID-19 on AMR and its management, respectively. A small proportion of physicians (pediatric, 10.6%; adult, 10%) perceived no impact of COVID-19 on the regional AMR landscape.

#### Factors impacting MDR infection management during the COVID-19 pandemic

*Which factors have impacted the treatment of MDR gram-negative bacterial infections in your hospital during the COVID-19 pandemic*? Physician responses: pediatric, 47 (94%); adult, 140 (90.9%).

According to most of the participants of the survey, the effective management of MDR gram-negative bacterial infections during the COVID-19 pandemic was significantly impacted by increased hospital occupancy, priorities shifting away from AMR surveillance, changing AMR epidemiology, and antibiotic shortages (S14 Table in S1 Appendix). However, 17 pediatric physicians and 25 adult physicians reported a lack of awareness and unavailability of data about the COVID-19 pandemic-associated factors that impacted the management of MDR gram-negative bacterial infections in the region.

#### Antibiotic use among COVID-19 patients

*How many of your COVID-19 patients were on antibiotics before your care and how many prescriptions were for presumed super imposed bacterial infections*? Physician responses: pediatric, 47 (94%); adult, 140 (90.9%).

**Fig 7:**
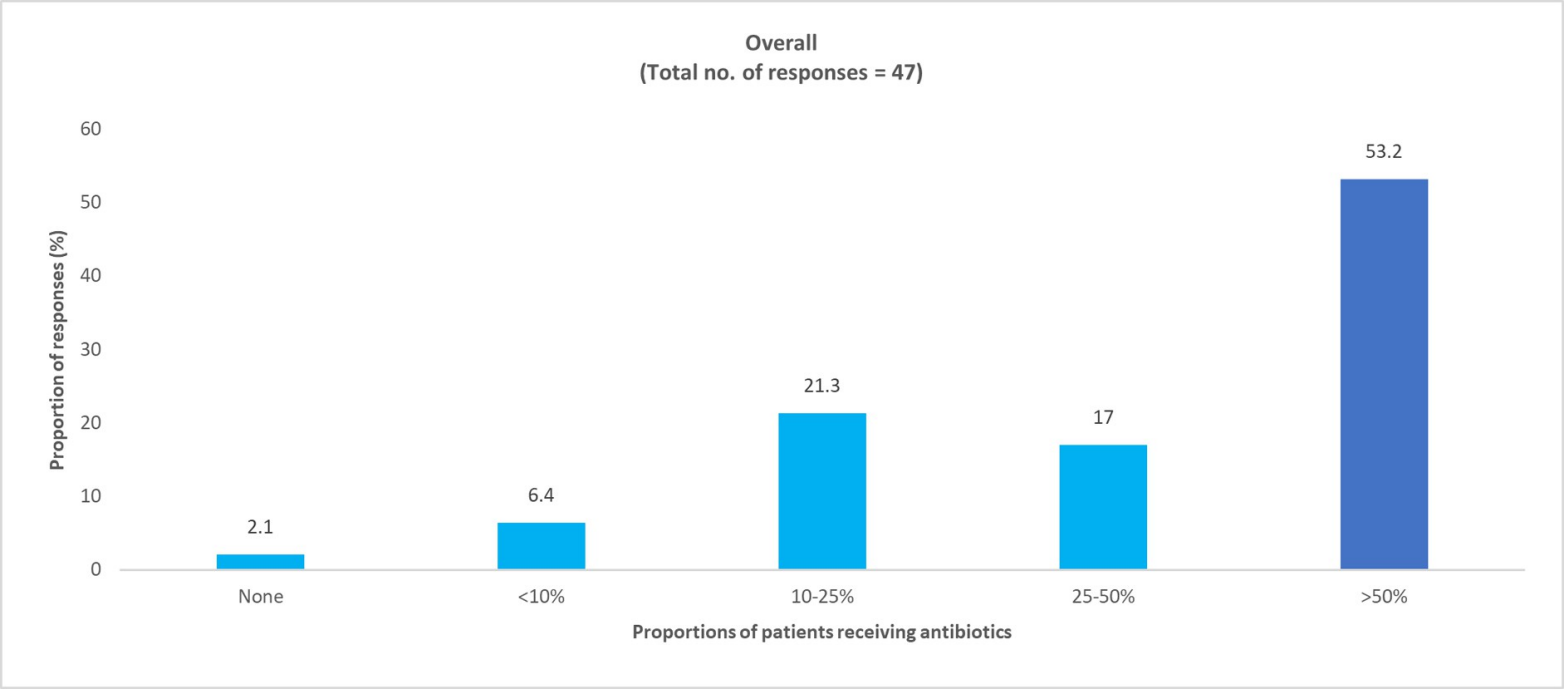
Physician perceived proportion of pediatric COVID-19 patients who had received antibiotics for presumed superimposed bacterial infections.

**Fig 8:**
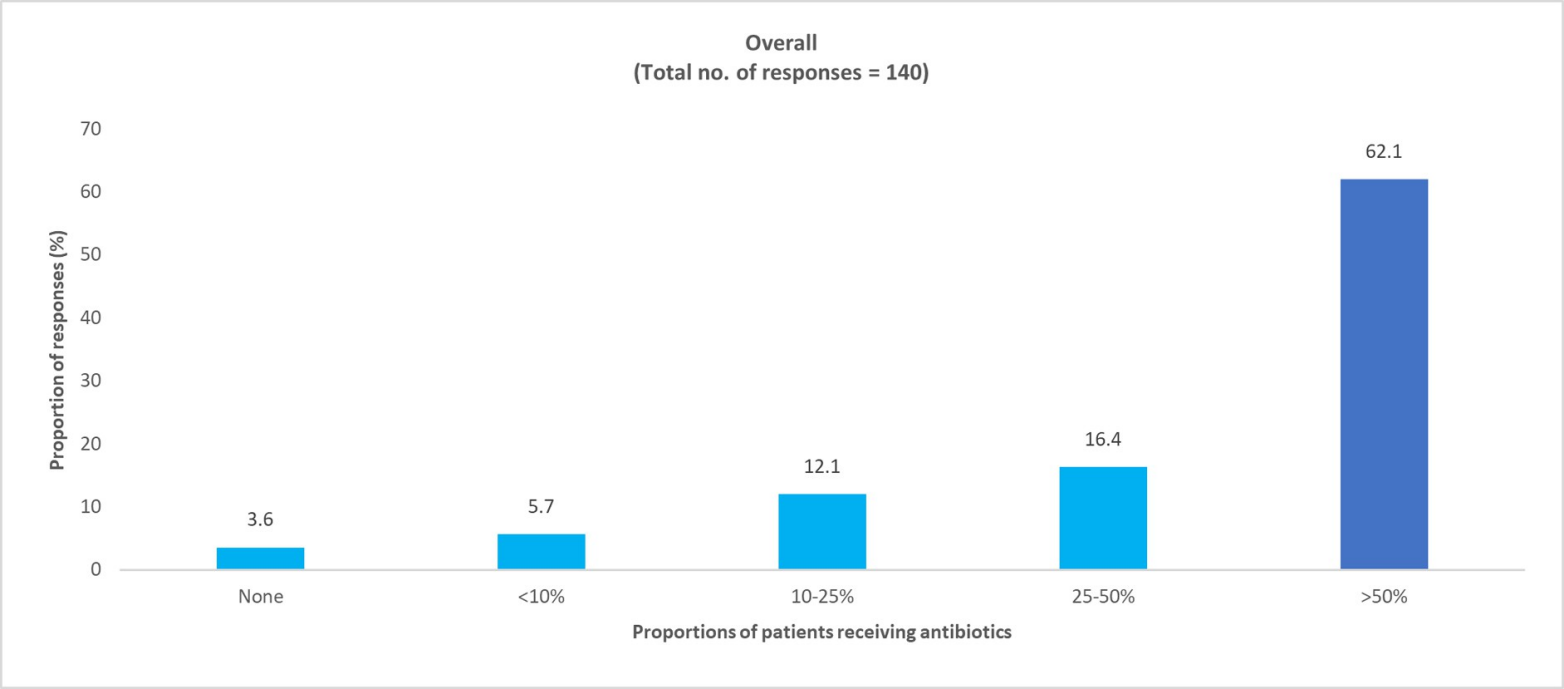
Physician perceived proportion of adult COVID-19 patients who had received antibiotics for presumed superimposed bacterial infections.

In the survey, most physicians reported that COVID-19 patients were already on antibiotics prior to consultation and a majority of them were receiving antibiotics for presumed superimposed bacterial infections (Figs 7 and 8 and S3 and S4 Figs in S1 Appendix).

#### MDR infections in COVID-19 ICU patients

*How frequently have you encountered MDR gram-negative bacteria in COVID ICU patients in your practice*? Physician responses: pediatric, 47 (94%); adult, 140 (90.9%).

**Fig 9:**
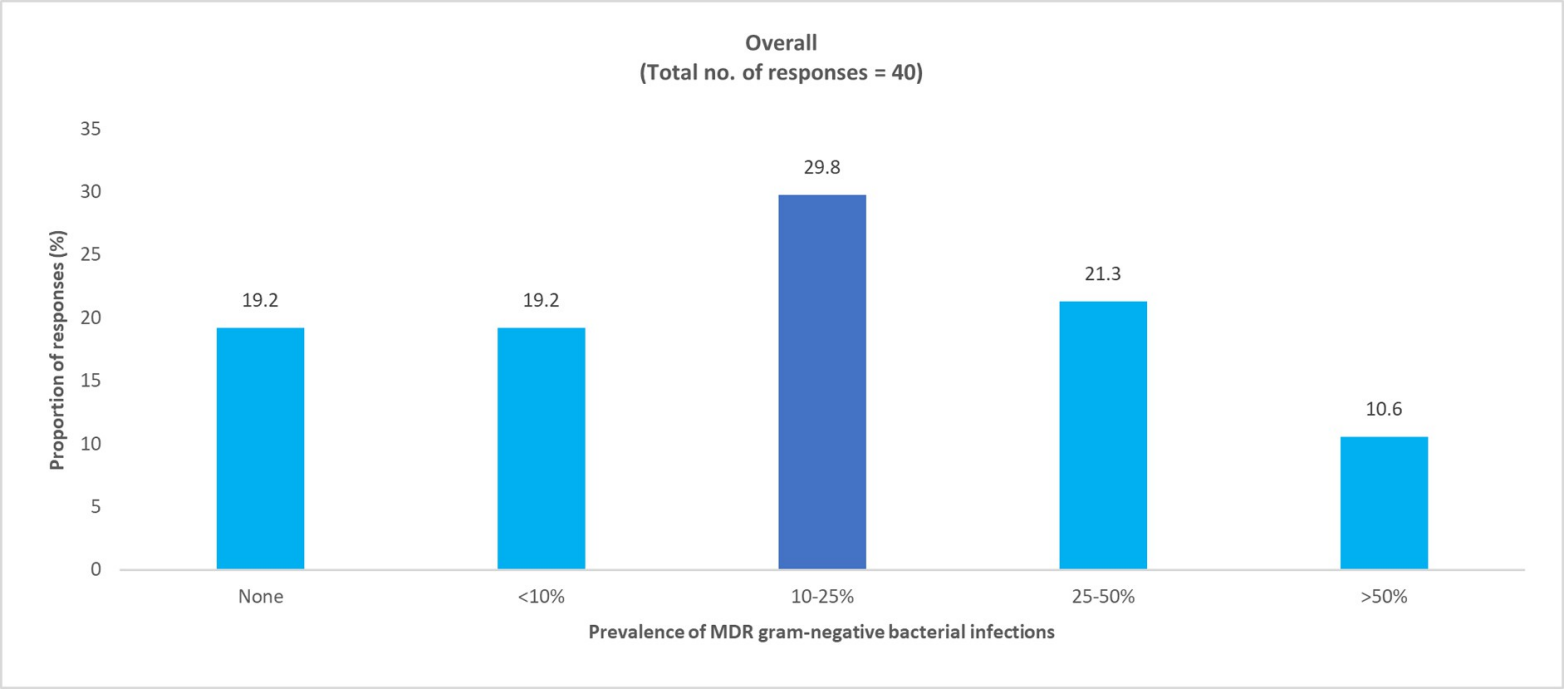
Physician-perceived prevalence of MDR gram-negative bacteria in pediatric COVID-19 ICU patients. ICU, intensive care unit; MDR, multidrug resistant.

**Fig 10:**
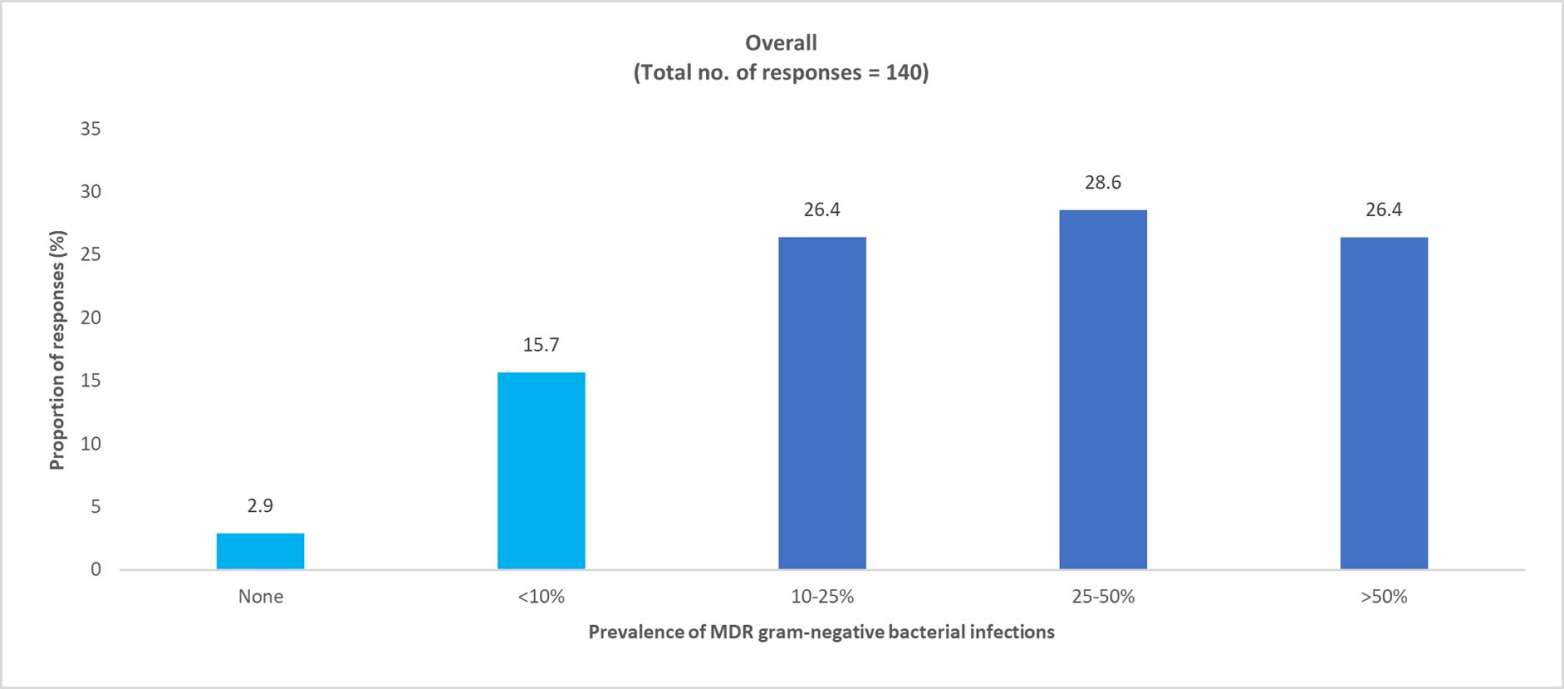
Physician-perceived prevalence of MDR gram-negative bacteria in adult COVID-19 ICU patients. ICU, intensive care unit; MDR, multidrug resistant.

Most physicians in the region reported that MDR gram-negative bacteria were identified in COVID-19 patients who were admitted in the ICU as shown in Figs 9 and 10 (S5 Fig in S1 Appendix).

### AMS initiatives in the region

#### Which of the following AMS structures are implemented in your institution?

Physician responses: pediatric, 46 (92%); adult, 138 (89.6%).

**Fig 11:**
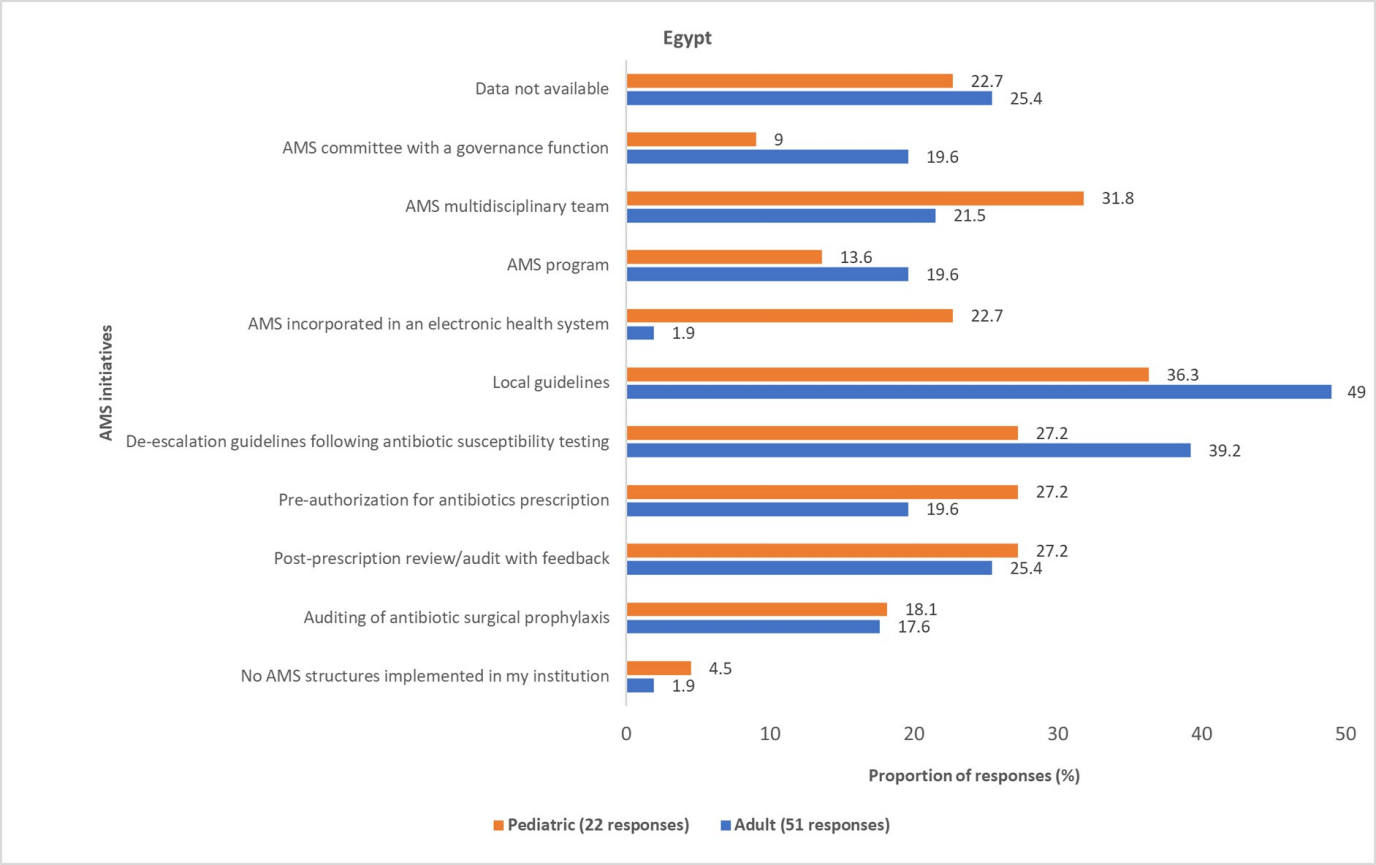
Physicians’ awareness of implemented AMS initiatives in Egypt. AMS, antimicrobial stewardship.

**Fig 12:**
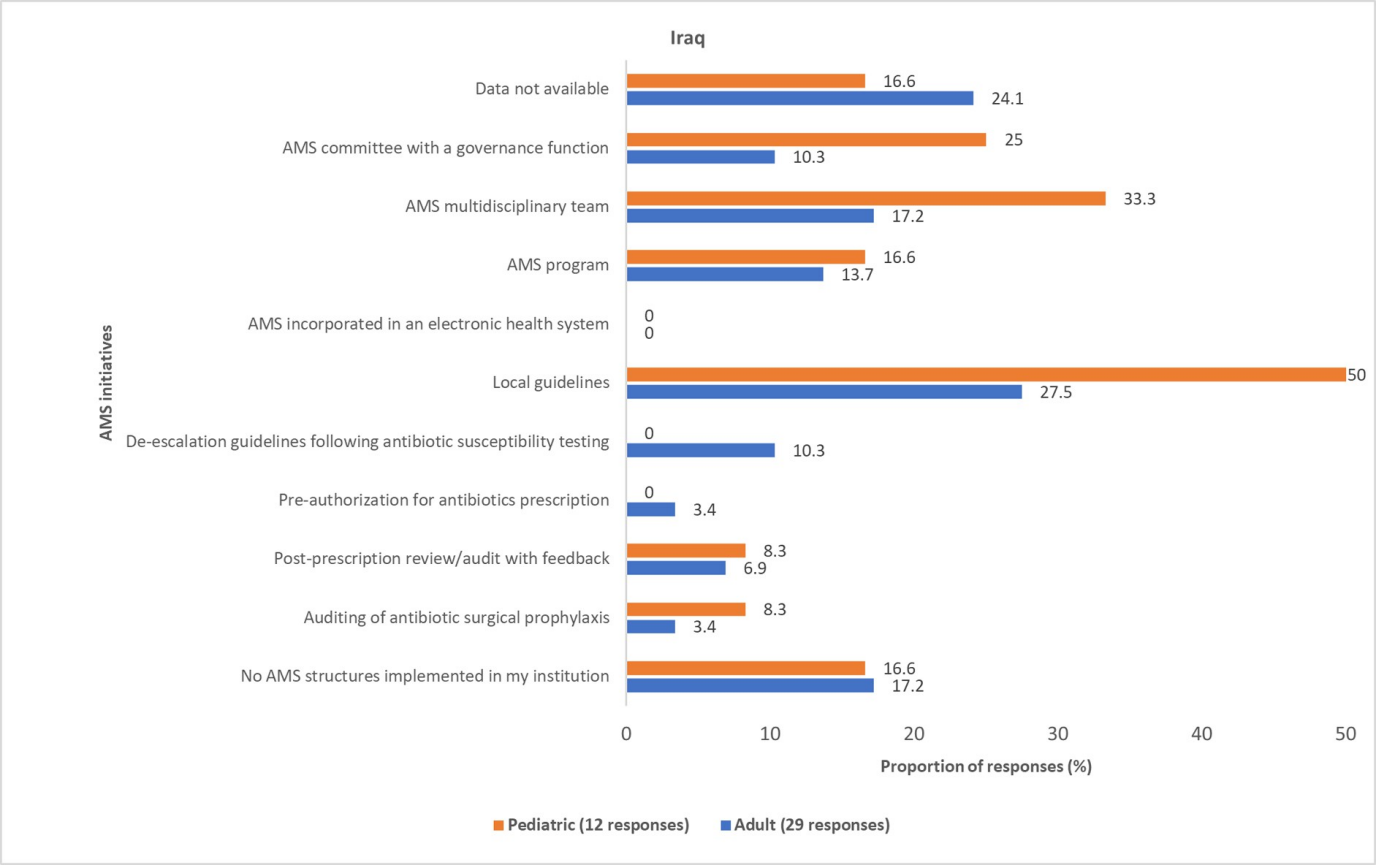
Physicians’ awareness of implemented AMS initiatives in Iraq. AMS, antimicrobial stewardship.

**Fig 13:**
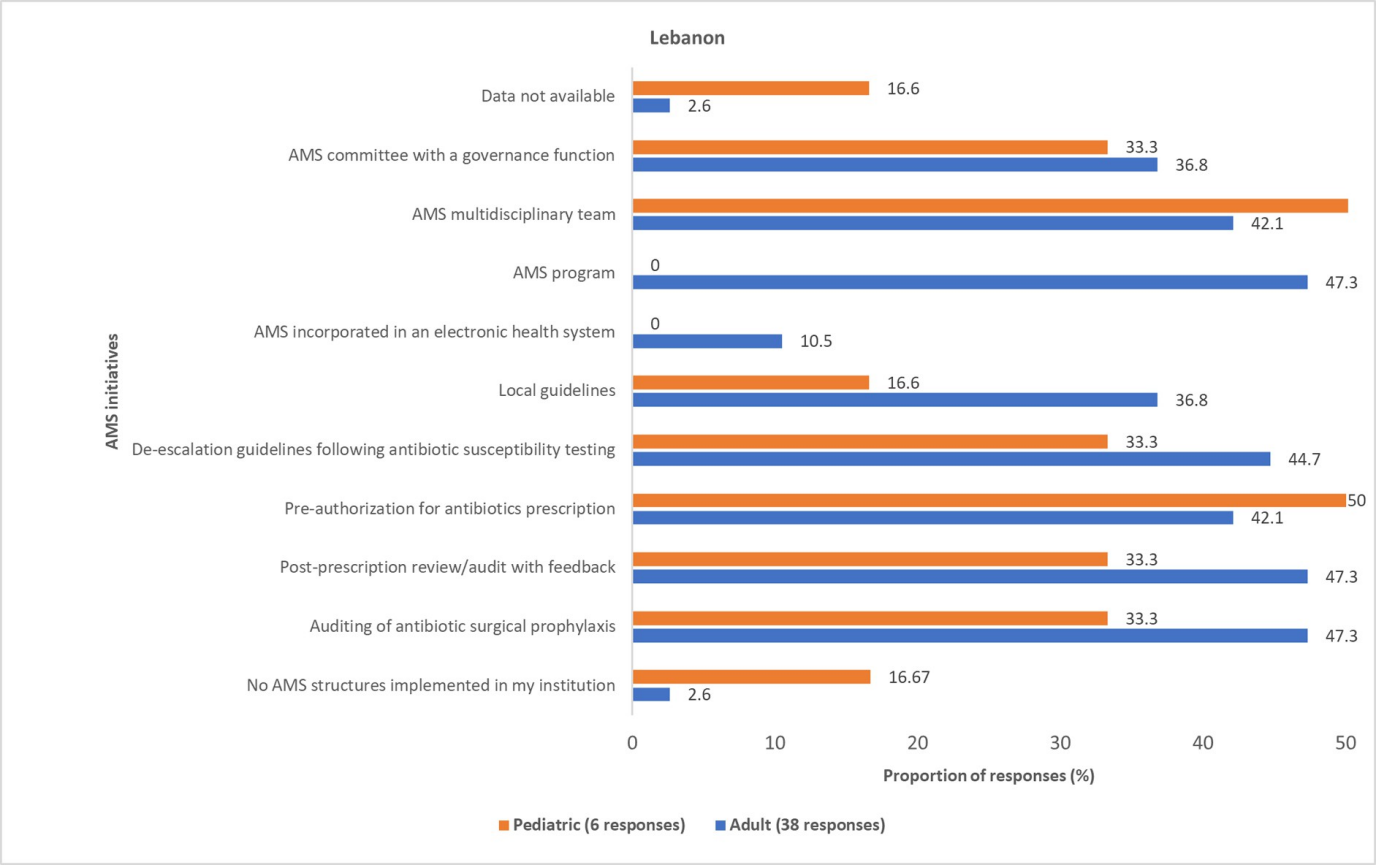
Physicians’ awareness of implemented AMS initiatives in Lebanon. AMS, antimicrobial stewardship.

**Fig 14:**
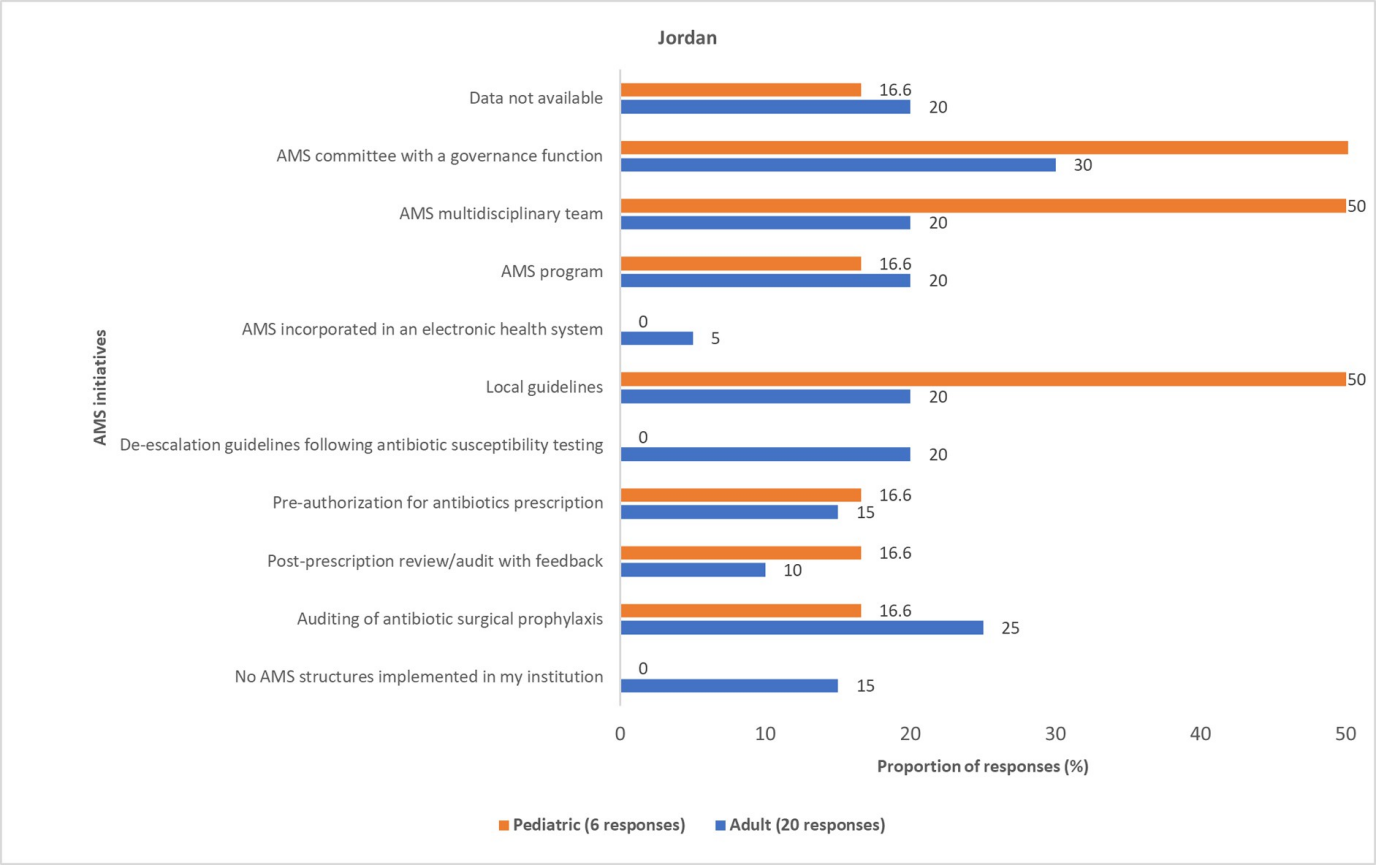
Physicians’ awareness of implemented AMS initiatives in Jordan. AMS, antimicrobial stewardship.

Most physicians reported various AMS initiatives for AMR governance and reporting that were implemented in hospitals across Egypt, Iraq, Jordan, and Lebanon (Figs 11–14 and S6 Fig in S1 Appendix). However, these initiatives are not standardized across the four countries. Some physicians (pediatric, 9 (19.5%); adult, 35 (25.3%)) reported that information about the AMS initiatives implemented in their respective institutions was unavailable to them.

#### Barriers to AMS implementation

*What are the main barriers to AMS implementation in your hospital*? Physician responses: pediatric, 42 (84%); adult, 133 (86.3%).

A lack of adequately trained multidisciplinary AMS staff, AMS knowledge, training gaps among physicians, and strained laboratory and financial resources were identified as the main barriers to AMS implementation across hospitals and institutions in the region (S15 Table in S1 Appendix). This was exacerbated by the lack of regional and national surveillance systems, as well as a general lack of commitment on the part of various key stakeholders toward AMS surveillance.

## Discussion

This survey provides a broad depiction of the scope of AMR-related challenges in Egypt, Iraq, Jordan, and Lebanon. As depicted in the survey, the participating physicians noted an upward trend in the prevalence of MDR gram-negative bacteria over the years, which has been exacerbated by the COVID-19 pandemic due to the unnecessary use of empirical antibiotics. The underdeveloped regional healthcare infrastructure, characterized by limited access to well-equipped laboratories and advanced diagnostic technologies compared to more developed nations, poses significant challenges for accurate microbiological diagnosis and AST. As a result, physicians heavily rely on a broad understanding of the general signs and symptoms of patients and the nature of antibiotics to inform the prescription of empirical therapies. Furthermore, there were notable variations in antibiotic prescription preferences among the physicians, which could be attributed to constraints such as limited access to targeted antibiotics, unavailability of diagnostic tools, and absence of standardized treatment protocols for antibiotic prescriptions. Interestingly, a considerable proportion of COVID-19 patients received antibiotics despite the absence of confirmed bacterial infections, indicating a potential issue of over-prescription. A general lack of awareness was identified among the participants about existing AMR governance and AMS initiatives in the region. This underscores the need to address specific areas, such as improving access to accurate diagnostic tools, optimizing antibiotic prescription practices, and increasing awareness about AMR governance and AMS initiatives, in order to effectively manage the increasing threat of AMR and promote prudent antibiotic use in the region.

AMR is a major concern for treating physicians because it severely limits the treatment options available to patients, leading to an increase in treatment costs and mortality [26, 27]. A thorough understanding of the local and regional epidemiology is necessary in order to efficiently manage MDR gram-negative bacterial infections [27]. However, the heterogeneity of the healthcare infrastructure and the varying economies in the region have limited the organized and standardized collation of AMR epidemiological data [26]. Research has attributed the high prevalence of AMR in Egypt, Iraq, Jordan, and Lebanon to the unnecessary use of antibiotics [27]. In order to efficiently manage MDR gram-negative bacterial infections, it is critical to consider the risk factors that contribute to the development and spread of AMR in the regional context. This survey discovered significant similarities in risk factors across the region which contributed to an increase in the prevalence of MDR gram-negative bacterial infections. However, there was a general lack of awareness about the prevalence and epidemiology of MDR gram-negative bacteria among physicians, indicating that information was not readily available.

In general, the region is facing resource and funding challenges as a result of the need for accelerated healthcare growth [28]. This lack of resources limits microbiological diagnostic capabilities in the region [27]. According to the survey, target organism genotyping and phenotyping were not routinely ordered for all patients prior to antibiotic prescription. Limited testing may have contributed to the gap in knowledge regarding AMR prevalence among physicians. The AMR landscape in the region is exacerbated by a general lack of surveillance, as there are no local or national registries or protocols for standardizing microbiological testing and AMR reporting [27]. Furthermore, this survey also highlights the diverse prescription practices among physicians in the region which can be attributed to the lack of standardized local prescription guidance or protocols.

Understanding the molecular characteristics of AMR patterns in MDR gram-negative bacterial infections throughout the region can help guide the use of novel antibiotics [27].

The nosocomial spread of MDR gram-negative bacteria has particularly increased during the COVID-19 pandemic [13, 14]. HCPs were found to be unaware of the impact of COVID-19 on AMR epidemiology and prevalence in the region. Due to higher hospital occupancy rates and a severe antibiotic shortage during the pandemic, managing MDR gram-negative bacterial infections became difficult, and patients were forced to be treated with broad-spectrum antibiotics despite more targeted antibiotics being indicated. Additionally, unregulated antibiotic sales have also significantly impacted AMR management [29].

AMS is an essential step in combating the growing threat of AMR by optimized use of antibiotics, improved microbiological laboratory capabilities, and regional and national initiatives for standardized AMR surveillance, microbiological testing, and AMR reporting [27]. Many countries in the region lack the necessary resources to establish an effective and dependable AMR surveillance system with rapid and accurate diagnostic tools [15, 16]. This survey highlighted a few local AMS initiatives that already exist in some institutions in Egypt, Iraq, Jordan, and Lebanon. However, due to fragmented healthcare infrastructure, limited resources, diverse local AMR management practices, and gaps in AMS knowledge and training among physicians, these initiatives are not standardized across the region. The difficulties in implementing standardized AMS initiatives have been exacerbated by a lack of commitment on the part of higher administration, which could be attributed to shifting priorities, a lack of available and trained personnel, and the enormous strain on the healthcare system as a result of the COVID-19 pandemic.

### Call to action

This survey provides useful insights about the perception and understanding of the barriers to effective AMR management and AMS implementation among physicians in Egypt, Iraq, Jordan, and Lebanon. The expert working group proposed a call to action based on the WHO AMS Toolkit in order to overcome these barriers and potentially serve as a guide for future AMR management and AMS implementation initiatives in the region [30]. The WHO proposed 5 key pillars of the AMS implementation package based on public health principles that aim to drive comprehensive and integrated AMS activities in health-care facilities (Table 3)

**Table 3:** Pillars of integrated AMS activities [30].

Pillar I	National AMS coordination mechanisms and guidelines
Pillar II	Antimicrobial access and regulation
Pillar III	Awareness, education, and training
Pillar IV	Water, sanitation and hygiene, and infection prevention and control
Pillar V	Surveillance, monitoring, and evaluation

AMS, antimicrobial stewardship.

According to the experts in the working group, it is crucial to consider the unique regional context during the implementation of AMS, including the regional healthcare infrastructure and the availability of resources such as funding and personnel. They emphasized the urgent need for collaborative efforts between the government, healthcare sector, pharmaceutical companies, and public health organizations to ensure swift, systematic, and standardized implementation of the WHO AMS Toolkit across the region.

It was noted that pharmaceutical companies and public health organizations can play a vital role in facilitating educational and training initiatives for communities and the healthcare fraternity, including pharmacists, and also provide technical assistance in regional data generation, research, and development of targeted novel antimicrobial agents. The experts called upon the governing authorities to develop policies that would facilitate the re-allocation of resources to improve access to antibiotics in the region and establish stringent regulations on the sale and consumption of antibiotics [20, 30, 31].

Moreover, collaborative efforts among stakeholders from all the relevant societal sectors, such as policy makers, decision makers, and healthcare administrators, are necessary to develop and implement standardized regional and national AMS protocols and guidelines [2, 30]. The experts recognized that the regional landscape for AMR surveillance and AMS governance is significantly underdeveloped, and immediate measures must be undertaken by national health authorities to enhance AMR reporting. This can provide crucial insights to international organizations that drive regional AMS initiatives. Egypt, Iraq, Jordan, and Lebanon are registered members of the WHO Global Antimicrobial Resistance and Use Surveillance System (GLASS) and continue to contribute towards the Global AMS initiative in varying capacities [30]. However, the existing AMS initiatives within the region must be strengthened to meet global standards, with a strong emphasis on optimizing antibiotic use based on the WHO’s Global Action Plan, involving patients, healthcare providers, pharmacists, and other relevant stakeholders in healthcare practice to support the regional AMR and AMS initiatives, and allocate the necessary resources to achieve the desired AMS targets.

## Conclusion

The management of MDR gram-negative bacterial infections is becoming increasingly complex, requiring a comprehensive understanding of the regional AMR epidemiology, appropriate antibiotic use, and efficient surveillance to identify and treat emerging AMR. This survey sheds light on the gaps in knowledge and awareness among physicians regarding AMR epidemiology and AMS initiatives, as well as the existing challenges related to the harmonization of AMR management and governance across Egypt, Iraq, Jordan, and Lebanon. The COVID-19 pandemic has compelled physicians to rethink current management strategies for MDR gram-negative bacterial infections in order to effectively address the alarmingly rising threat of AMR.

To tackle this issue, regional AMS measures, guidelines, and policies must be developed and implemented using a phased, interdisciplinary approach in collaboration with all the key stakeholders. This calls for leadership, dedication, faith, empowerment, education, resources, and efforts at all societal levels. There is also a pressing need for qualified AMS personnel as well as pediatric and adult infectious disease specialists in these countries. While the findings of this survey provide subjective insights of physicians into the general AMR and AMS landscape in the region, future studies should also evaluate the understanding of AMR and AMS among patients in the region.

### Limitations of the study

The survey participants included chest physicians/pulmonologists, infectious disease specialists, intensivists, microbiologists, neonatologists, and pediatricians from Egypt, Iraq, Jordan, and Lebanon as they were more frequently involved in AMS initiatives. Other specialties like hematologists, transplant physicians/surgeons, or clinical pharmacists were not included. Their opinions and insights on AMR and AMS were, therefore, unavailable. The number of completed responses received was low and variable across the four countries, which may have hindered our analysis. The information on the prevalence of MDR gram-negative bacterial infections is based on the personal perceptions of the participating physicians and carries a risk of bias and must be interpreted with caution. Furthermore, the survey did not evaluate the specific protocols, guidelines, or standards that were used to inform the genotyping, phenotyping, and AST practices in the region.

## Supporting information

S1 Appendix Supplementary information. (PDF)

S2 Appendix Survey questionnaire template. (PDF)
