# Supplementary material for: Antimicrobial resistance landscape and COVID-19 impact in Egypt, Iraq, Jordan, and Lebanon: A survey-based study and expert opinion
Source: PLoS One. 2023 Jul 27;18(7):e0288550. doi: 10.1371/journal.pone.0288550 (PMC10374138; doi:10.1371/journal.pone.0288550)
Supplement: S1 Appendix — (PDF) [file pone.0288550.s001.pdf]

AMR Survey Supplementary Tables and Figures

Table of Contents

Supplementary Tables **2**
Supplementary Table 1. Survey participation, response rates, and specialty by country. 2
Supplementary Table 2. Survey respondents' specialty by country (n). 2
Supplementary Table 3. Physician perception of common risk factors for resistant gram-negative bacterial infections. 2
Supplementary Table 4. Physician perception of infections in which gram-negative bacteria were frequently encountered. 3
Supplementary Table 5. Physician-perceived prevalent AMR mechanisms in hospital-acquired MDR gram-negative bacterial infections. 4
Supplementary Table 6. Physician perception of commonly identified gram-negative bacteria in hospital-acquired infections and resistance enzyme coding gene mutations. 4
Supplementary Table 10. Preferred choice of antibiotic for the treatment of pediatric MDR gram-negative bacterial infections across the region as reported by the survey participants. 7
Supplementary Table 11. Preferred choice of antibiotic for the treatment of pediatric MDR gram-negative bacterial infections by country as reported by the survey participants. 8
Supplementary Table 11. Preferred choice of antibiotic for the treatment of adult MDR gram-negative bacterial infections by country as reported by the survey participants. 9
Supplementary Table 13. Physician perception of the factors impacting the treatment of MDR gram-negative bacterial infections during the COVID-19 pandemic. 10
Supplementary figures **12**
Supplementary Figure 1. Physician perception of the prevalence of gram-negative bacteria in various infection types. 12
Supplementary Figure 2. Physician perceived prevalence of AMR enzyme-coding gene mutations in gram-negative bacteria. 14
Supplementary Figure 3. Antibiotic use among COVID-19 patients before meeting the physician. 18
Supplementary Figure 4. Antibiotic use among COVID-19 patients for presumed superimposed bacterial infections. 19
Supplementary Figure 5. Physician perception of the prevalence of multidrug-resistant gram-negative bacteria in COVID intensive-care unit patients. 19
Supplementary Figure 6. Implemented AMS initiatives. 20

Supplementary Tables

Supplementary Table 1. Survey participation, response rates, and specialty by country.

Country	Distribution (N)	Responses (n)	Response rate (%)
Egypt	171	82	47.9
Iraq	106	43	40.5
Jordan	108	30	27.7
Lebanon	162	49	30.2
**Total:**	**547**	**204**	**37.2**

Supplementary Table 2. Survey respondents' specialty by country (n).

Specialty	Egypt	Iraq	Jordan	Lebanon	Total
**Pediatric physicians**					
Neonatologist	4	2	0	1	7
Pediatric infectious disease specialist	2	0	1	2	5
Pediatric intensivist	3	0	0	2	5
Pediatrician	9	8	2	2	21
Total					38
**Adult physicians**					
Chest physician/pulmonologist	5	7	7	5	24
Infectious disease specialist	4	2	4	30	40
Intensivist	30	7	4	6	47
Microbiologist	18	0	3	0	21
Total					132
Other*	7	17	9	1	34
**Total number of respondents:**	**82**	**43**	**30**	**49**	**204**

**Specialty not reported.*

Supplementary Table 3. Physician perception of common risk factors for resistant gram-negative bacterial infections.

Common risk factors for MDR gram-negative bacterial infections	Responses				
	Overall (%)	Egypt (%)	Iraq (%)	Jordan (%)	Lebanon (%)
Pediatric	***N=48***	***n=23***	***n=12***	***n=5***	***n=8***
Prior infection with MDR gram-negative bacteria	**5.3**	5.3	5	5	5.8
Colonization with MDR gram-negative bacteria	**5.2**	5.1	5	5.5	5.6
Long stay at a healthcare facility (e.g., hospital, nursing home or long-term acute care facility)	**4.7**	4.7	4.7	4.6	4.6
Prior treatment with antibiotics within the last 90 days (3 months)	**4.1**	4.2	4	4.4	3.5
Travel or residence in areas with high rates of MDR organisms	**2.6**	2.7	5	2	2.6
Other	**1.3**	1.3	1.2	2	1
Adult	***N=152***	***n=57***	***n=30***	***n=24***	***n=41***
Prior infection with MDR gram-negative bacteria	**6.2**	6	5.9	6	6.8
Colonization with MDR gram-negative bacteria	**6**	5.8	6.1	5.9	6.3
Long stay at a healthcare facility (e.g., hospital, nursing home or long-term acute care facility)	**5.5**	5.5	5.4	6	5.1
Prior treatment with antibiotics within the last 90 days (3 months)	**5.4**	5.7	5.4	4.9	5.4
Travel or residence in areas with high rates of MDR organisms	**3.7**	3.3	4	3.6	3.9
The patient is a health-care professional	**2.5**	2.7	2.5	2.2	2.4
Other	**1.3**	1.7	1.1	1	1

**Common risk factors for carbapenem-resistant gram-negative bacterial infections**	**Responses**				
	**Overall (%)**	**Egypt (%)**	**Iraq (%)**	**Jordan (%)**	**Lebanon (%)**
Pediatric	***N=49***	***n=24***	***n=12***	***n=5***	***n=8***
Previous antibiotic use	8.7	8.6	10.3	8.7	6.6
Previous carbapenem use	9	9.3	9.1	7	8.6
Previous colonization	7.9	7.3	8.6	9	8.2
Mechanical ventilation	7.3	7.2	7.2	9	7.5
Previous intensive care unit stay	7.5	7.6	6.4	9	8.5
Dialysis	5.2	4.7	5.9	6.2	4.7
Central lines and catheters	6.7	6.8	6.5	6	6.7
Length of stay in hospital	6.8	7.1	6.4	9	6.2
≥2 Comorbidities	5	5.4	4.3	2	5.5
APACHE SCORE II	2.8	3.2	1.9	5.6	2.2
Other	1.5	1.6	1.8	1	1
Adult	***N=145***	***n=56***	***n=26***	***n=23***	***n=40***
Previous antibiotic use	8.5	8.5	9.2	7.9	8.3
Previous carbapenem use	9.4	9.6	8.6	8.5	10.1
Previous colonization	7.9	7.3	8.1	7.5	8.7
Mechanical ventilation	7.5	7.6	7.2	7.8	7.3
Previous intensive care unit stay	7.4	7.7	6	7.2	7.7
Dialysis	4.9	4.5	5.2	5.8	4.6
Central lines and catheters	5.5	5.1	5.5	6.7	5.4
Length of stay in hospital	6.4	6.5	6.2	6.8	6
≥2 Comorbidities	4.3	4.6	4.7	4.1	3.8
APACHE SCORE II	3.7	3.7	4.8	3.6	3.2
Other	1.8	1.9	2.8	1.1	1.3

MDR, multidrug-resistant.

Supplementary Table 4. Physician perception of infections in which gram-negative bacteria were frequently encountered.

**Infection type**	**Responses**				
	**Overall (%)**	**Egypt (%)**	**Iraq (%)**	**Jordan (%)**	**Lebanon (%)**
Pediatric	***N=48***	***n=23***	***n=12***	***n=5***	***n=8***
Lower respiratory tract infection (hospital-acquired pneumonia/ventilator-associated pneumonia)	**5.1**	5.4	5.2	4	5
Skin and soft tissue infection	**3.2**	3.2	3.8	3.7	2.2
Complicated urinary tract infection	**4**	3.5	3.8	5	4.8
Catheter-related bloodstream infection	**4.5**	4.6	4.3	3.7	4.7
Complicated intraabdominal infection	**3.4**	3.8	2.6	4	3.1
Others	**1.1**	1.2	1.2	1	1
Adult	***N=153***	***n=58***	***n=30***	***n=24***	***n=41***
Lower respiratory tract infection (hospital-acquired pneumonia/ventilator-associated pneumonia)	**4.5**	4.6	4.1	4.6	4.5
Skin and soft tissue infection	**2.3**	2.3	3.1	2	1.8
Complicated urinary tract infection	**3.3**	3	2.8	3.4	3.8
Catheter-related bloodstream infection	**2.7**	2.7	2.6	2.8	2.6
Complicated intraabdominal infection	**2.3**	2.4	2.3	2.2	2.2

Supplementary Table 5. Physician-perceived prevalent AMR mechanisms in hospital-acquired MDR gram-negative bacterial infections.

AMR Mechanisms	Responses (n)	Egypt (%)	Iraq (%)	Jordan (%)	Lebanon (%)
**Pediatric**					
ESBL-producing *Acinetobacter baumannii*	**26**	43.6	34.7	65.7	47.9
Carbapenem-resistant *Acinetobacter baumannii*	**26**	46.6	39.2	58	44.1
ESBL-producing *Escherichia coli*	**27**	49.4	38.2	51.7	39.5
Carbapenem-resistant *Escherichia coli*	**27**	44.6	32.8	25	14.4
ESBL-producing *Klebsiella pneumoniae*	**26**	51.9	38	37	35.8
Carbapenem-resistant *Klebsiella pneumoniae*	**24**	50.5	34.2	29.7	13.4
Carbapenem-resistant *Pseudomonas aeruginosa*	**24**	41.4	34.2	36.7	29.3
MDR *Pseudomonas aeruginosa*	**25**	38.2	30.7	32.3	22.7
Other *Enterobacteriaceae*	**22**	35.6	33.7	45	19
Other carbapenem-resistant *Enterobacteriaceae*	**21**	33.9	30	37.3	6.2
**Adult**					
ESBL-producing *Acinetobacter baumannii*	**84**	47.2	39	34.3	55.4
Carbapenem-resistant *Acinetobacter baumannii*	**86**	57.9	37.6	58	59.7
ESBL-producing *Escherichia coli*	**90**	45.5	45.7	43.1	42
Carbapenem-resistant *Escherichia coli*	**85**	40	40.8	23.2	11.4
ESBL-producing *Klebsiella pneumoniae*	**93**	55.8	41.7	30.2	37.5
Carbapenem-resistant *Klebsiella pneumoniae*	**87**	54.2	36.7	26.5	14
Carbapenem-resistant *Pseudomonas aeruginosa*	**91**	47.4	48.1	31.4	25.8
MDR *Pseudomonas aeruginosa*	**86**	48.5	47.2	31.2	18
Other *Enterobacteriaceae*	**81**	35.9	30.6	24.1	23.7
Other carbapenem-resistant *Enterobacteriaceae*	**79**	33.1	31	21.5	11.7

AMR, antimicrobial resistance; ESBL, extended-spectrum β-lactamase; MDR, multi-drug-resistant.

Supplementary Table 6. Physician perception of commonly identified gram-negative bacteria in hospital-acquired infections and resistance enzyme coding gene mutations.

Commonly identified gram-negative bacteria in hospital-acquired infections	Responses				
	Overall (%)	Egypt (%)	Iraq (%)	Jordan (%)	Lebanon (%)
Pediatric	***N=50***	***n=24***	***n=12***	***n=6***	***n=8***
Acinetobacter baumannii	**2.6**	3.1	2.7	2.6	1.4
Escherichia coli	**3.9**	3.2	4.1	5	4.6
Klebsiella pneumoniae	**4**	4.4	3.8	3.4	3.8
Pseudomonas aeruginosa	**3**	3.3	2.2	3.2	3.2
Other *Enterobacteriaceae*	**1.7**	1.4	2.2	1.6	1.8
Adult	***N=146***	***n=57***	***n=27***	***n=23***	***n=39***
Acinetobacter baumannii	**2.9**	3.1	3	3	2.3
Escherichia coli	**3.6**	2.9	3.3	3.9	4.4
Klebsiella pneumoniae	**4**	4.6	3.6	3.3	3.5
Pseudomonas aeruginosa	**3.1**	3	3.3	3.3	3
Other *Enterobacteriaceae*	**1.4**	1.14	1.5	1.4	1.6
**Commonly identified antimicrobial resistance enzyme coding gene mutations in gram-negative bacteria**	**Responses**				
	**Overall (%)**	**Egypt (%)**	**Iraq (%)**	**Jordan (%)**	**Lebanon (%)**
Pediatric	***N=50***	***n=24***	***n=12***	***n=6***	***n=8***
Extended-spectrum β-lactamases (TEM, SHV, CTX-M)	**30**	29.1	8.3	50	50
Carbapenemase blaOXA-48	**16**	20.8	8.3	16.6	12.5
Carbapenemase blaKPC	**10**	8.3	0	0	37.5
Carbapenemase bla MBLs (NDM and VIM)	**14**	16.6	8.3	0	25
AmpC beta-lactamases	**6**	12.5	0	0	0
mcr-1 (colistin resistance)	**4**	4.1	0	16.6	0

Others	**6**	4.1	8.3	0	12.5
Information not available	**58**	50	83.3	50	50
Adult	***N=154***	***n=58***	***n=31***	***n=24***	***n=41***
Extended-spectrum β-lactamases (TEM, SHV, CTX-M)	**37**	39.6	3.2	50	51.2
Carbapenemase blaOXA-48	**18.1**	25.8	12.9	8.3	17
Carbapenemase blaKPC	**5.8**	6.9	3.2	12.5	2.4
Carbapenemase bla MBLs (NDM and VIM)	**16.2**	32.7	6.4	8.3	4.8
AmpC beta-lactamases	**15.5**	8.6	0	20.8	34.1
mcr-1 (colistin resistance)	**3.9**	0	3.2	12.5	4.8
Others	**1.9**	0	0	12.5	0
Data not available	**53.2**	46.5	83.8	41.6	46.3

Supplementary Table 7. Physician awareness of genotyping and phenotyping practices of target organisms.

**Is the genotyping and phenotyping of target organisms performed in your institution?**	**Responses**				
	**Overall (%)**	**Egypt (%)**	**Iraq (%)**	**Jordan (%)**	**Lebanon (%)**
Pediatric	***N=47***	***n=22***	***n=12***	***n=5***	***n=7***
Yes	**14.8**	22.7	0	0	28.5
No	**72.3**	72.7	0.7	66.6	71.4
I don't know	**12.7**	4.5	0.2	33.3	0
Adult	***N=143***	***n=53***	***n=30***	***n=21***	***n=39***
Yes	**28.6**	41.5	16.6	23.8	23
No	**51.7**	37.7	66.6	42.8	64.1
I don't know	**19.5**	20.7	16.6	33.3	12.8

Supplementary Table 8. Physician perception regarding routine antimicrobial susceptibility testing practices.

**Barriers to routine antimicrobial susceptibility testing**	**Responses**				
	**Overall (%)**	**Egypt (%)**	**Iraq (%)**	**Jordan (%)**	**Lebanon (%)**
Pediatric	***N=45***	***n=21***	***n=11***	***n=6***	***n=7***
Antimicrobial sensitivity testing technology limitations	**6.4**	6.8	7.1	4.8	5.5
Lack of micro lab capacity (this includes trained microbiologists and resources)	**6**	5.9	7	6.5	4.5
Lack of adequate sampling	**4.8**	4.3	5.8	4.8	4.7
Lack of timely reporting	**4.9**	5.2	4.7	4.5	5
Absence of robust communication policies	**4.4**	4.7	4.1	4.5	4
Costs of tests	**4.7**	4.6	4	5.1	5.8
Other	**2.4**	2.5	2	1.7	3.2
There are no barriers	**2.6**	3.2	1.3	3	3
Adult	***N=133***	***n=51***	***n=29***	***n=20***	***n=33***
Antimicrobial sensitivity testing technology limitations	**6.4**	6.3	6.7	7	6.1
Lack of micro lab capacity (this includes trained microbiologists and resources)	**6**	5.7	6.9	5.7	5.8
Lack of adequate sampling	**5.3**	4.8	5.8	5.8	5
Lack of timely reporting	**4.9**	5.3	4.5	5	4.7
Absence of robust communication policies	**4.7**	5.2	4.3	3.7	4.5
Costs of tests	**5.1**	5.4	4.1	5	5.6
Other	**2.2**	2.2	2	2.4	2.2
There are no barriers	**2.5**	1.8	1.8	3.3	3.7
**Ordering microbial culture and antimicrobial susceptibility testing**	**Responses**				
	**Overall (%)**	**Egypt (%)**	**Iraq (%)**	**Jordan (%)**	**Lebanon (%)**
Pediatric	***N=46***	***n=22***	***n=12***	***n=6***	***n=6***
Test all infected intensive care unit patients	**26**	27.2	16.6	33.3	33.3
Test only patients at high risk of MDR infection	**13**	13.6	25	0	0

Test only patients failing initial therapy	**8.7**	13.6	8.3	0	0
Test all patients requiring antibiotic treatment	**45.6**	40.9	33.3	66.6	66.6
No antibiotic susceptibility testing is available	**2.1**	0	8.3	0	0
Other	**4.3**	4.5	8.3	0	0
Adult	***N=138***	***n=51***	***n=29***	***n=20***	***n=38***
Test all infected intensive care unit patients	**47.8**	74.5	27.5	35	34.2
Test only patients at high risk of MDR infection	**6.5**	0	13.7	10	7.8
Test only patients failing initial therapy	**10.1**	3.9	34.4	10	0
Test all patients requiring antibiotic treatment	**31.8**	21.5	13.7	35	57.8
No antibiotic susceptibility testing is available	**1.4**	0	6.9	0	0
Other	**2.1**	0	3.4	10	0

MDR, multidrug resistant.

Supplementary Table 9. Physician perception regarding the medical management of MDR gram-negative bacterial infections.

**Barriers to successful medical management of MDR gram-negative bacterial infections**	**Responses**				
	**Overall (%)**	**Egypt (%)**	**Iraq (%)**	**Jordan (%)**	**Lebanon (%)**
Pediatric	***N=46***	***n=21***	***n=12***	***n=6***	***n=7***
Limited microbiological diagnostic capabilities	**6.1**	6.6	6	6	5.2
Availability and cost of newer antibiotics	**5.4**	5.1	5.2	6	6
The policies and practices inside the hospital	**4.6**	5.4	4.5	3.6	3.8
Lack of knowledge of hospital or national epidemiology and lack of national guidelines	**4.6**	4.4	5	4.8	4.4
Administrative challenges	**3.2**	3	3	3.1	4.4
Lack of an infectious diseases specialist	**3.1**	3.2	3.9	2.6	2.2
Other	**1.1**	1.1	1	1	1.7
Adult	***N=135***	***n=50***	***n=28***	***n=20***	***n=37***
Limited microbiological diagnostic capabilities	**5.5**	5.1	6	5.6	5.5
Availability and cost of newer antibiotics	**5.6**	5.4	4.7	6	6.4
The policies and practices inside the hospital	**4.6**	5	4.1	4.6	4.4
Lack of knowledge of hospital or national epidemiology and lack of national guidelines	**4.3**	4.3	4.8	4.1	4.1
Administrative challenges	**3.5**	3.7	3	2.9	3.8
Lack of an infectious diseases specialist	**3.2**	3.6	3.7	3.4	2
Other	**1.5**	1.3	1.8	1.4	1.6
**Factors informing the choice of empirical antibiotic therapy**	**Responses**				
	**Overall (%)**	**Egypt (%)**	**Iraq (%)**	**Jordan (%)**	**Lebanon (%)**
Pediatric	***N=46***	***n=22***	***n=11***	***n=6***	***n=7***
Antibiotic spectrum	**7.7**	7.6	8.1	8	7
Site of infection	**7.6**	7.6	7.4	7.6	7.8
Comorbidities	**5.4**	5	6	5	5.7
Antibiotic cost	**3.8**	3.2	5.5	3.3	2.8
Antibiotic safety	**5.4**	4.9	5.3	7	5.5
Pharmacokinetics/pharmacodynamics	**3.8**	3.7	4	2.5	4.4
Drug-drug interaction	**3.3**	3.5	3.8	3	2.2
History of previous antibiotic exposure within last 3 months	**4.3**	3.1	3.1	4.8	4.1
Published guidelines and local antibiograms	**5**	6	2	7	5.1
Adult	***N=131***	***n=48***	***n=27***	***n=20***	***n=36***
Antibiotic spectrum	**7.7**	7.5	8	7.3	8
Site of infection	**7.3**	7.1	7.2	7.4	7.4
Comorbidities	**5.5**	5.6	6	5.1	5.2
Antibiotic cost	**3.4**	3	4.5	3.7	3

Antibiotic safety	**4.7**	4.8	5.1	4.6	4.5
Pharmacokinetics/pharmacodynamics	**4**	4.3	3.6	3.4	4.2
Drug-drug interaction	**3.4**	3.3	4	3.5	3
History of previous antibiotic exposure within last 3 months	**4.5**	4.3	3.3	5	5.4
Published guidelines and local antibiograms	**5.1**	6	4.2	5.1	4.5

MDR, multidrug resistant.

Supplementary Table 10. Preferred choice of antibiotic for the treatment of pediatric MDR gram-negative bacterial infections across the region as reported by the survey participants.

Antibiotic	Pediatric			Adult		
	First-line	Second-line	Third-line	First-line	Second-line	Third-line
**Number of responses**	**47**	**47**	**47**	**143**	**143**	**143**
**Severe ESBL-producing *Enterobacteriaceae* infections**						
New BL-BLI[a] (%)	14.8	19.1	NA	31.4	30	NA
Conventional BL-BLI[b] (%)	23.4	6.3		20.9	6.2	
Aminoglycoside (%)	12.7	19.1		0.7	6.2	
Carbapenem (%)	46.1	25.5		41.2	29.3	
Polymyxin (%)	0	0		2.8	4.9	
Fluroquinolone (%)	0	2.1		0.7	6.2	
Monobactam (%)	0	0		0.7	0	
Aminoglycoside combinations (%)	NA	21.2		NA	14.6	
Other combinations (%)	NA	6.3		NA	NA	
Other antibiotics (%)	2.1	0		1.4	2.1	
**Severe carbapenem-resistant *Enterobacteriaceae* infections**						
New BL-BLI[a] (%)	27.6	21.2	19.1	46.1	23	23.7
Aminoglycoside (%)	14.8	6.3	6.3	4.2	8.3	2.8
Tigecycline (%)	NA	NA	NA	3.5	10.4	6.2
Polymyxin (%)	12.7	6.3	6.3	11.8	14.6	14.6
Fosfomycin (%)	0	4.2	8.5	1.4	0.7	2.8
Aminoglycoside combinations (%)	23.4	31.9	21.2	9.7	17.4	13.9
Polymyxin combinations (%)	12.7	12.7	8.5	15.3	15.3	17.4
Other combinations (%)	4.2	17	21.2	4.2	6.2	11.1
Other antibiotics (%)	4.2	0	8.5	3.5	3.5	6.9
**Severe MDR *Pseudomonas aeruginosa* infections**						
Polymyxin (%)	2.1	0	8.5	10.4	8.3	11.1
Carbapenem (%)	23.4	14.8	8.5	17.4	6.9	3.5
Other classical anti-pseudomonal β-lactam antibiotics[c] (%)	23.4	19.1	8.5	19.5	14.6	12.5
Ceftazidime-avibactam (%)	6.3	14.8	14.8	15.3	18.8	17.4
Ceftolozane-tazobactam (%)	0	6.3	4.2	8.3	6.9	6.9
Colistin with carbapenem/aminoglycoside combination (%)	25.5	23.4	19.1	16.7	23	18.8
Aminoglycoside with carbapenem combination (%)	8.5	14.8	6.3	7.6	13.2	11.1
Fosfomycin with carbapenem/β-lactam/ aminoglycoside/colistin combination (%)	6.3	2.1	12.7	0.7	3.5	11.8
Other antibiotics (%)	4.2	4.2	17	3.5	4.2	6.2

BL-BLI, β-lactam/β-lactamase inhibitor; ESBL, extended-spectrum β-lactamase; MDR, multidrug-resistant; NA, not applicable. [a]New BL-BLI include ceftazidime-avibactam and ceftolozane-tazobactam; [b]Examples of conventional BL-BLI include piperacillin-tazobactam, amoxicillin-clavulanic acid, and ampicillin-sulbactam; [c]Other classical anti-pseudomonal β-lactam antibiotics include aztreonam, cefepime, ceftazidime, and piperacillin-tazobactam. Note: 'Not applicable' implies that a drug(s) or combination(s) was not considered in a particular line of treatment.

Supplementary Table 11. Preferred choice of antibiotic for the treatment of pediatric MDR gram-negative bacterial infections by country as reported by the survey participants.

**Antimicrobial treatment**	**Antimicrobial treatment by country**											
	**Egypt**			**Iraq**			**Jordan**			**Lebanon**		
	**First-line**	**Second-line**	**Third-line**	**First-line**	**Second-line**	**Third-line**	**First-line**	**Second-line**	**Third-line**	**First-line**	**Second-line**	**Third-line**
**Number of responses**	**22**			**12**			**6**			**7**		
**Severe ESBL-producing *Enterobacteriaceae* infections**												
Polymyxin (%)	0	0	NA	0	0	NA	0	0	NA	0	0	NA
Fluroquinolone (%)	0	0		0	8.3		0	0		0	0	
Monobactam (%)	0	0		0	0		0	0		0	0	
Other combinations (%)	NA	13.6		NA	0		NA	0		NA	0	
Other antibiotics (%)	0	0		0	0		16.6	0		0	0	
**Severe carbapenem-resistant *Enterobacteriaceae* infections**												
Aminoglycoside (%)	4.5	4.5	0	33.3	0	16.6	0	16.6	0	0	14.2	14.2
Fosfomycin (%)	0	4.5	9	0	0	8.3	0	0	16.6	0	14.2	0
Other combinations (%)	9	18.1	18.1	0	25	16.6	0	16.6	33.3	28.5	0	28.5
Other antibiotics (%)	0	0	4.5	8.3	0	16.6	0	0	0	42.8	0	14.2
**Severe MDR *Pseudomonas aeruginosa* infections**												
Polymyxin (%)	4.5	0	9	0	0	0	0	0	0	0	0	28.5
Ceftolozane-tazobactam (%)	0	9	4.5	0	8.33	0	0	0	16.6	0	0	0
Fosfomycin with carbapenem/β-lactam/ aminoglycoside/colistin combination (%)	0	0	13.6	16.6	0	8.3	50	0	0	14.2	14.2	28.5
Other antibiotics (%)	0	4.5	18.1	8.3	8.3	25	0	0	0	14.2	0	14.2

ESBL, extended-spectrum β-lactamase; MDR, multidrug-resistant. Note: 'Not applicable' implies that a drug(s) or combination(s) was not considered in a particular line of treatment

Supplementary Table 12. Preferred choice of antibiotic for the treatment of adult MDR gram-negative bacterial infections by country as reported by the survey participants.

Antimicrobial treatment	Antimicrobial treatment by country											
	Egypt			Iraq			Jordan			Lebanon		
	First-line	Second-line	Third-line	First-line	Second-line	Third-line	First-line	Second-line	Third-line	First-line	Second-line	Third-line
**Number of responses**	**53**			**30**			**21**			**39**		
**Severe ESBL-producing *Enterobacteriaceae* infections**												
Polymyxin (%)	7.5	9.4	NA	0	0	NA	0	9.5	NA	0	0	NA
Fluroquinolone (%)	0	3.7		3.3	20		0	4.7		0	0	
Monobactam (%)	0	0		0	0		4.7	0		0	0	
Other antibiotics (%)	1.8	3.7		0	0		0	0		2.5	2.5	
**Severe carbapenem-resistant *Enterobacteriaceae* infections**												
Fosfomycin (%)	0	0	0	6.6	3.3	3.3	0	0	4.7	0	0	2.5
Other combinations (%)	3.7	7.5	7.5	10	13.3	23.3	4.7	0	0	0	2.5	5.1
Other antibiotics (%)	3.7	3.7	3.7	6.6	6.6	3.3	0	0	14.2	2.5	2.5	2.5
**Severe MDR *Pseudomonas aeruginosa* infections**												
Ceftolozane-tazobactam (%)	1.8	7.5	3.7	3.3	3.3	10	4.7	0	4.7	23	12.8	10.2
Fosfomycin with carbapenem/β-lactam/ aminoglycoside/colistin combination (%)	0	3.7	15	3.3	3.3	16.6	0	4.7	4.7	0	2.5	7.6
Other antibiotics (%)	3.7	3.7	5.6	3.3	6.6	6.6	4.7	4.7	4.7	2.5	2.5	7.6

ESBL, extended-spectrum β-lactamase; MDR, multidrug-resistant; NA, not applicable. Note: 'Not applicable' implies that a drug(s) or combination(s) was not considered in a particular line of treatment.

Supplementary Table 13. Physician awareness and perception of the impact of COVID-19 on the AMR landscape.

Impact of COVID-19 on the AMR landscape	Responses				
	**Overall (%)**	**Egypt (%)**	**Iraq (%)**	**Jordan (%)**	**Lebanon (%)**
Pediatric	***N=47***	***n=22***	***n=12***	***n=6***	***n=7***
Yes	**63.8**	59	75	50	71.4
No	**14.8**	13.6	8.3	16.6	28.5
I don't know	**21.2**	27.2	16.6	33.3	0
Adult	***N=140***	***n=51***	***n=30***	***n=21***	***n=39***
Yes	**80**	78.4	83.3	85	76.9
No	**7.8**	9.8	0	5	12.8
I don't know	**12.1**	11.7	16.6	10	10.2
**Impact of COVID-19 on bacterial epidemiology**	**Responses**				
	**Overall (%)**	**Egypt (%)**	**Iraq (%)**	**Jordan (%)**	**Lebanon (%)**
Pediatric	***N=47***	***n=22***	***n=12***	***n=6***	***n=7***
Yes	**55.3**	50	75	33.3	57.1
Yes, only in the intensive care unit	**14.8**	9	8.3	33.3	28.5
No	**17**	13.6	16.6	33.3	14.2
I don't know	**12.7**	27.2	0	0	0
Adult	***N=140***	***n=51***	***n=30***	***n=20***	***n=39***
Yes	**55.7**	41.1	70	65	58.9
Yes, only in the intensive care unit	**17.1**	27.4	13.3	15	7.6
No	**11.4**	13.7	3.3	10	15.3
I don't know	**15.7**	17.6	13.3	10	17.9
**Increased AMR in patients with active/past COVID-19 infection**	**Responses**				
	**Overall (%)**	**Egypt (%)**	**Iraq (%)**	**Jordan (%)**	**Lebanon (%)**
Pediatric	***N=47***	***n=22***	***n=12***	***n=6***	***n=7***
Yes	**38.3**	45.4	16.6	16.6	71.4
No	**10.6**	9	16.6	16.6	0
Data not available	**51**	45.4	66.6	66.6	28.5
Adult	***N=140***	***n=51***	***n=30***	***n=20***	***n=39***
Yes	**68.5**	74.5	66.6	70	61.5
No	**10**	3.9	3.3	10	23
Data not available	**21.4**	21.5	30	20	15.3

AMR, antimicrobial resistance.

Supplementary Table 14. Physician perception of the factors impacting the treatment of MDR gram-negative bacterial infections during the COVID-19 pandemic.

Factors impacting treatment of MDR gram-negative bacterial infections during COVID-19	Responses				
	**Overall (%)**	**Egypt (%)**	**Iraq (%)**	**Jordan (%)**	**Lebanon (%)**
Pediatric	***N=47***	***n=22***	***n=12***	***n=6***	***n=7***
Interrupted supply chains, drug shortages or stock outs	**19.1**	13.6	25	0	42.8
Changes in AMR epidemiology	**14.8**	13.6	8.3	33.3	14.2
Deprioritisation of AMR surveillance and AMS	**12.7**	13.6	8.3	16.6	14.2
Full hospital occupancy	**12.7**	4.5	16.6	16.6	28.5
All of the above	**23.4**	13.6	33.3	0	57.1
I don't know	**36.1**	40	50	33.3	0
None of the above	**10.6**	18.1	0	16.6	0

Adult	***N=143***	***n=51***	***n=30***	***n=20***	***n=39***
Interrupted supply chains, drug shortages or stock outs	**24.2**	17.6	16.6	30	35.9
Changes in AMR epidemiology	**27.8**	25.4	16.6	50	28.2
Deprioritisation of AMR surveillance and AMS	**16.4**	9.8	13.3	20	25.6
Full hospital occupancy	**15.7**	11.7	20	20	15.3
All of the above	**30.7**	25.4	40	30	30.7
Data not available	**17.8**	29.4	13.3	20	5.1
None of the above	**10**	9.8	0	5	20.5

AMR, antimicrobial resistance; AMS, antimicrobial stewardship; MDR, multidrug resistance.

Supplementary Table 15. Physician perception of the main barriers to AMS implementation.

**Main barriers to AMS implementation**	**Responses**				
	**Overall (%)**	**Egypt (%)**	**Iraq (%)**	**Jordan (%)**	**Lebanon (%)**
Pediatric	***N=42***	***n=21***	***n=10***	***n=5***	***n=6***
Lack of adequate staff for AMS	**7.9**	8.1	8	8	7.2
Lack of education and training of healthcare facility staff on AMS	**7.7**	8.1	7.5	7	7.8
Lack of laboratory resources to culture and test isolates for drug susceptibility	**6.9**	6.7	7.9	6.2	6.2
Absence of adequate funding for development of infrastructure	**6.9**	6.8	7.1	5.8	8.4
Lack of regional and national surveillance systems	**6.3**	6.1	6.8	5.8	6.8
Lack of automated system to track antibiotic prescriptions and AMR surveillance	**5.6**	5.1	5.6	7.4	5.6
Miscommunication between different specialties	**4.9**	5.1	4.5	5.4	4.8
Poor understanding of AMS among HCPs	**4.2**	4.6	3.9	4.4	3.6
Lack of commitment of higher administration	**3.6**	4.5	2.5	3.2	3.6
None of the above	**1.5**	1.6	1.1	1	2.5
Adult	***N=133***	***n=48***	***n=29***	***n=20***	***n=36***
Lack of adequate staff for AMS	**9.6**	9.9	9.8	10.2	8.6
Lack of education and training of healthcare facility staff on AMS	**9**	9.2	9.5	9	8.2
Lack of laboratory resources to culture and test isolates for drug susceptibility	**7.9**	7.8	8.8	8	7.2
Absence of adequate funding for development of infrastructure	**7.7**	7.3	7.7	7.7	8.5
Lack of regional and national surveillance systems	**7.3**	7.3	6.9	7	7.8
Lack of automated system to track antibiotic prescriptions and AMR surveillance	**6.4**	6.5	6	6.2	7
Miscommunication between different specialties	**6**	6.2	5.5	5.7	6.2
Poor understanding of AMS among HCPs	**5.4**	5.5	5	5.2	5.8
Lack of commitment of higher administration	**4.3**	4	3.8	4.6	4.9
None of the above	**2.8**	2.6	2.9	2	3.3
Data not available	**1.9**	1.8	1.8	1.6	2.5

Supplementary figures

Supplementary Fig 1. Physician perception of the prevalence of gram-negative bacteria in various infection types.

Pediatric

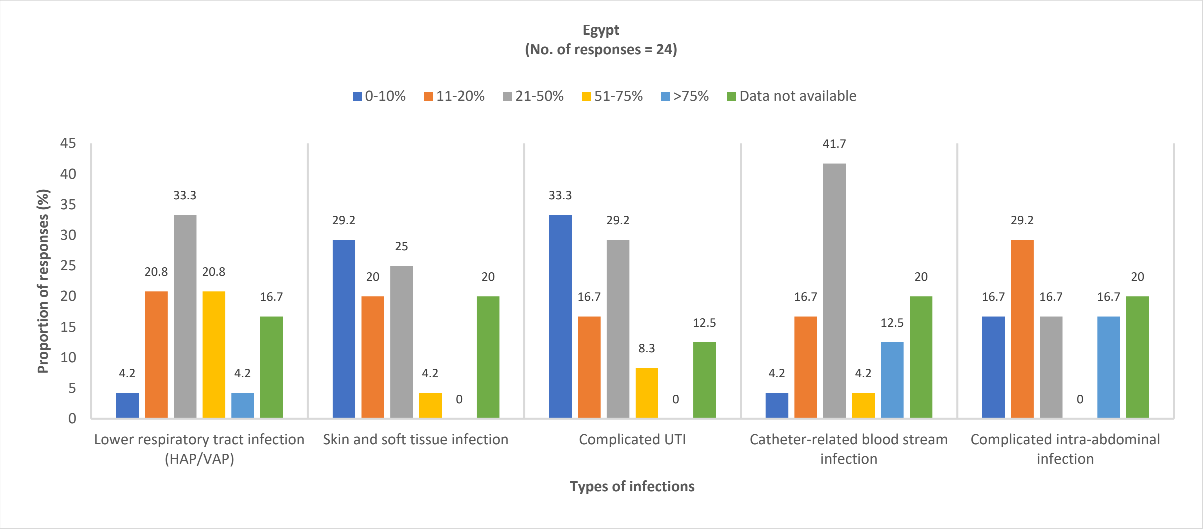

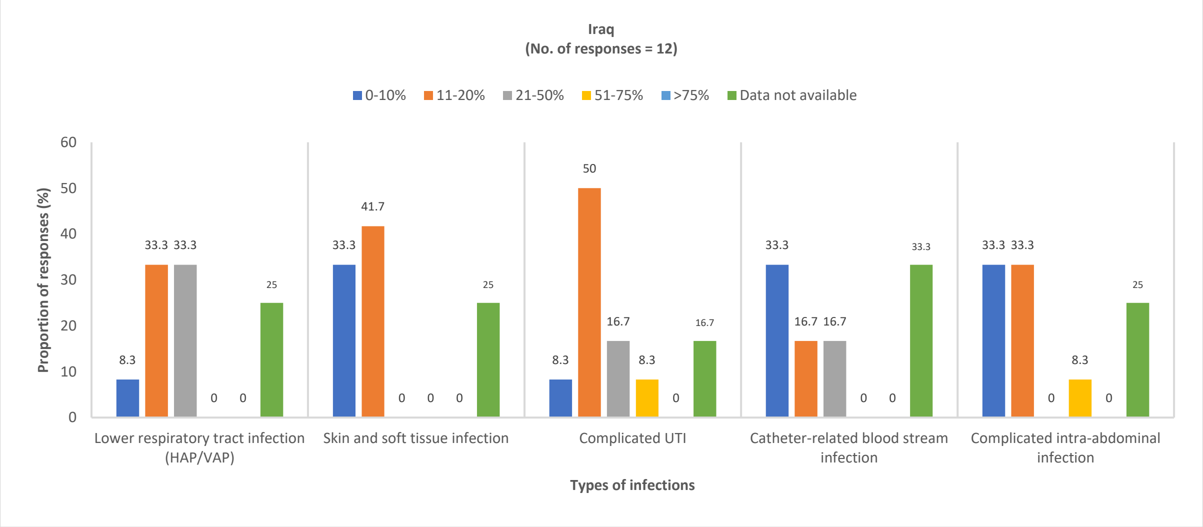

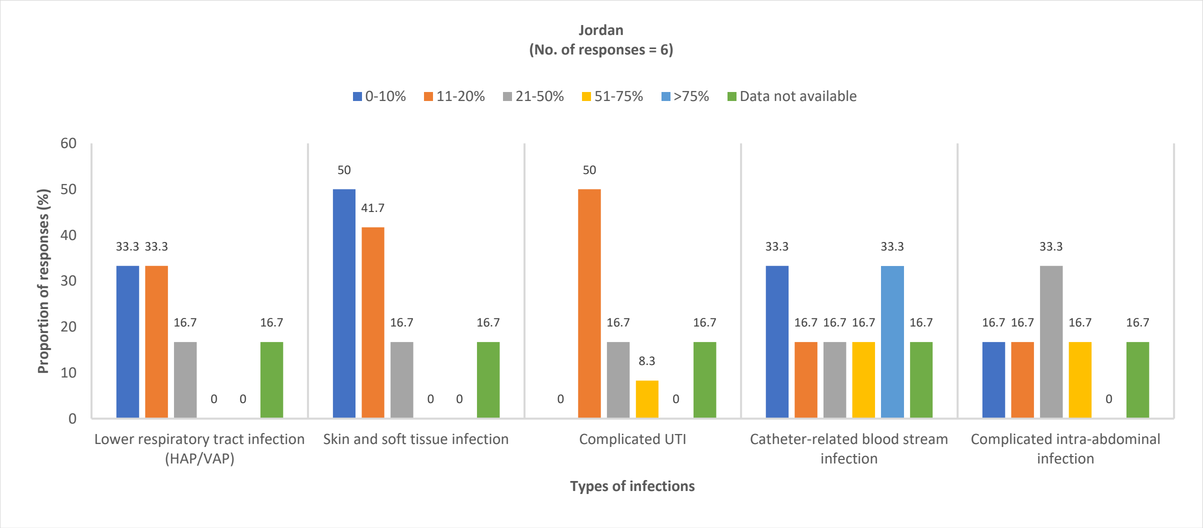

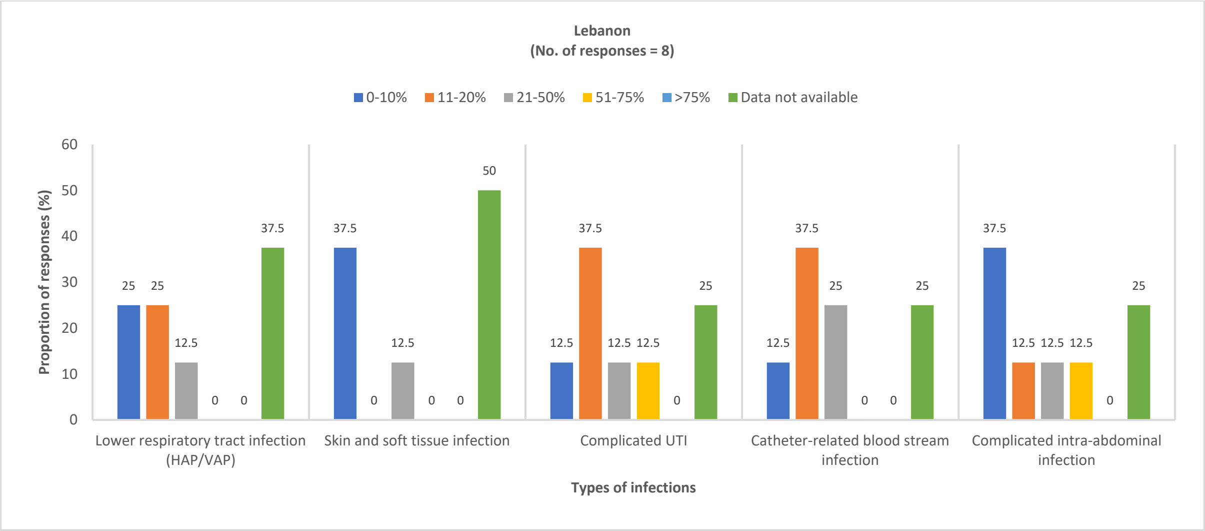
Lebanon
(No. of responses = 8)
0-10%
11-20%
21-50%
51-75%
>75%
Data not available
Proportion of responses (%)
Lower respiratory tract infection (HAP/VAP)
Skin and soft tissue infection
Complicated UTI
Catheter-related blood stream infection
Complicated intra-abdominal infection
Types of infections

Adult

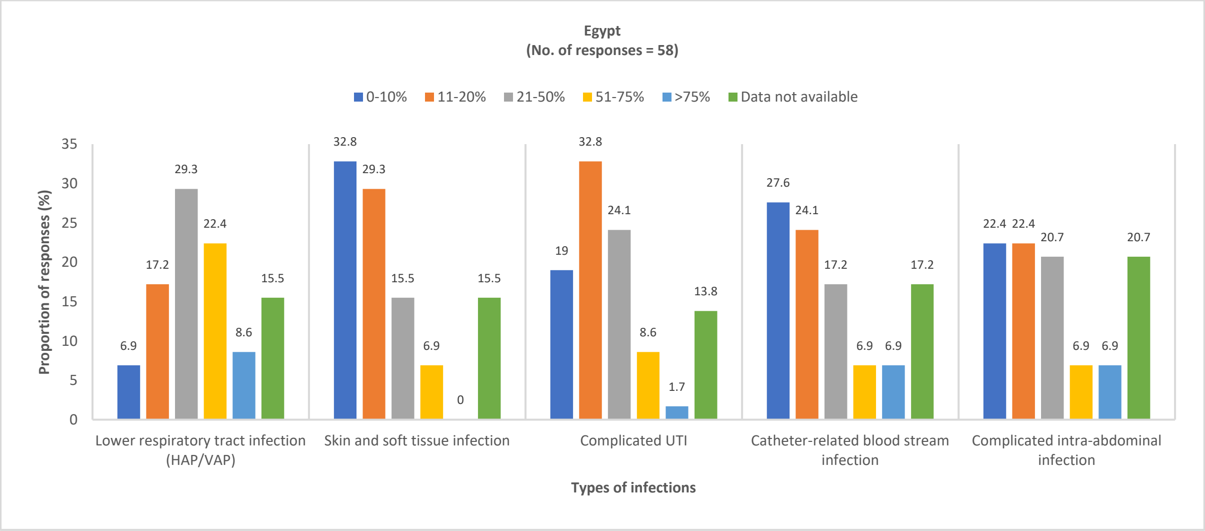
Egypt
(No. of responses = 58)
0-10%
11-20%
21-50%
51-75%
>75%
Data not available
Proportion of responses (%)
Lower respiratory tract infection (HAP/VAP)
Skin and soft tissue infection
Complicated UTI
Catheter-related blood stream infection
Complicated intra-abdominal infection
Types of infections

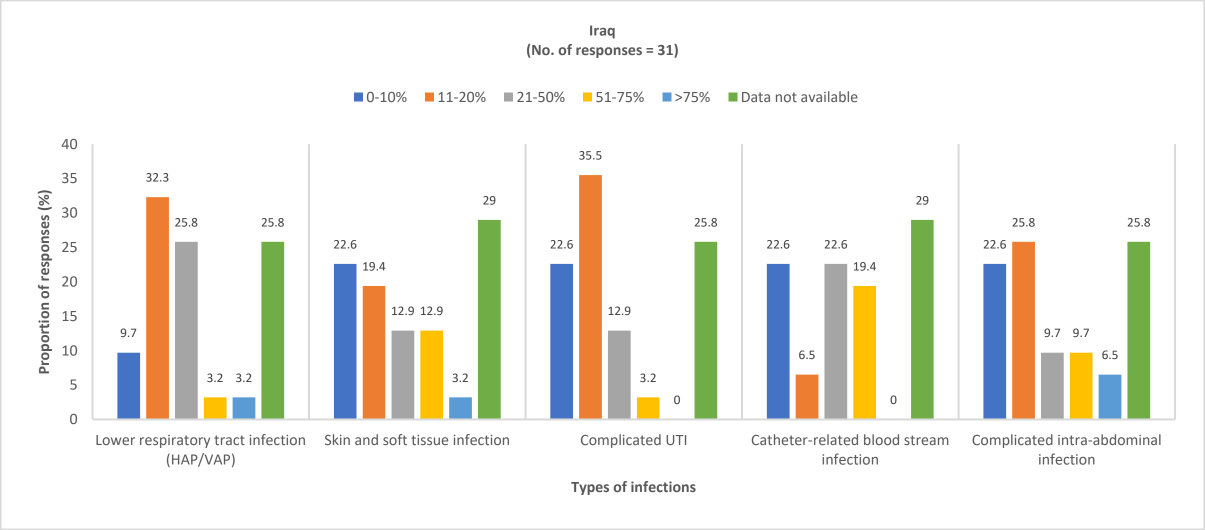
Iraq
(No. of responses = 31)
0-10%
11-20%
21-50%
51-75%
>75%
Data not available
Proportion of responses (%)
Lower respiratory tract infection (HAP/VAP)
Skin and soft tissue infection
Complicated UTI
Catheter-related blood stream infection
Complicated intra-abdominal infection
Types of infections

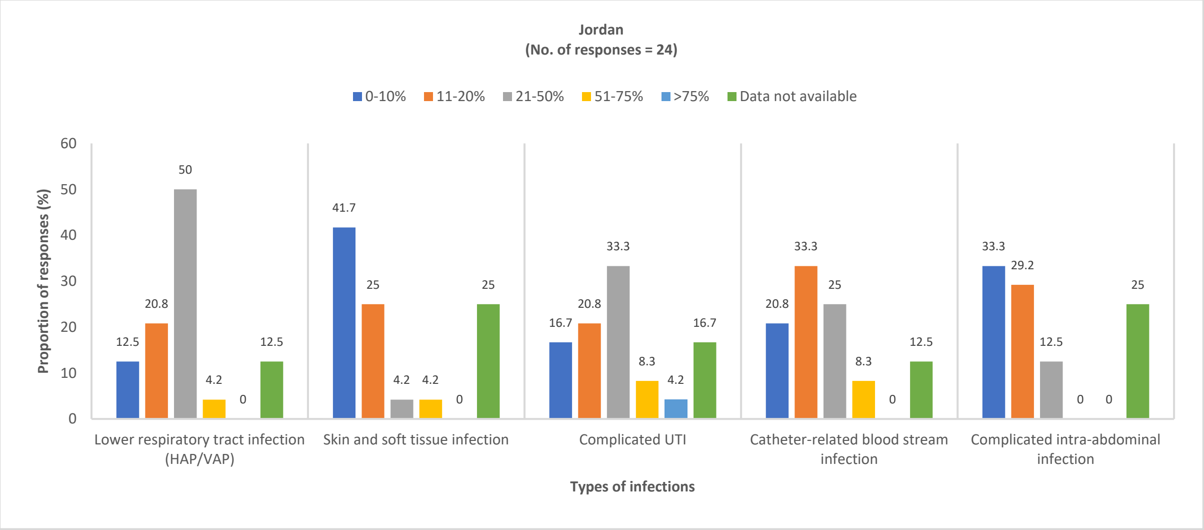

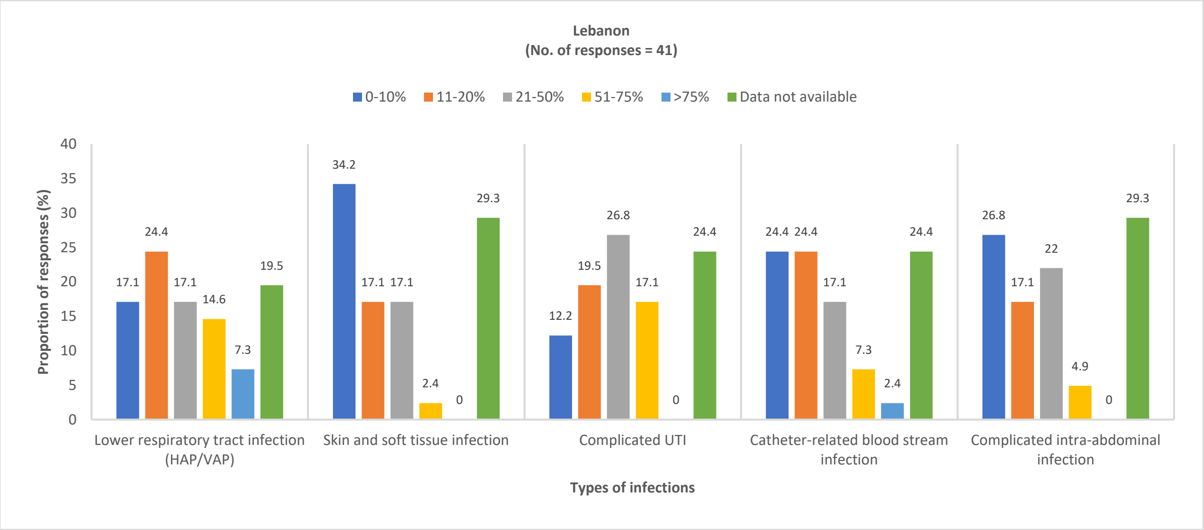

HAP, hospital-acquired pneumonia; UTI, urinary tract infections; VAP, ventilator-acquired pneumonia.

Supplementary Fig 2. Physician perceived prevalence of AMR enzyme-coding gene mutations in gram-negative bacteria.

Pediatric

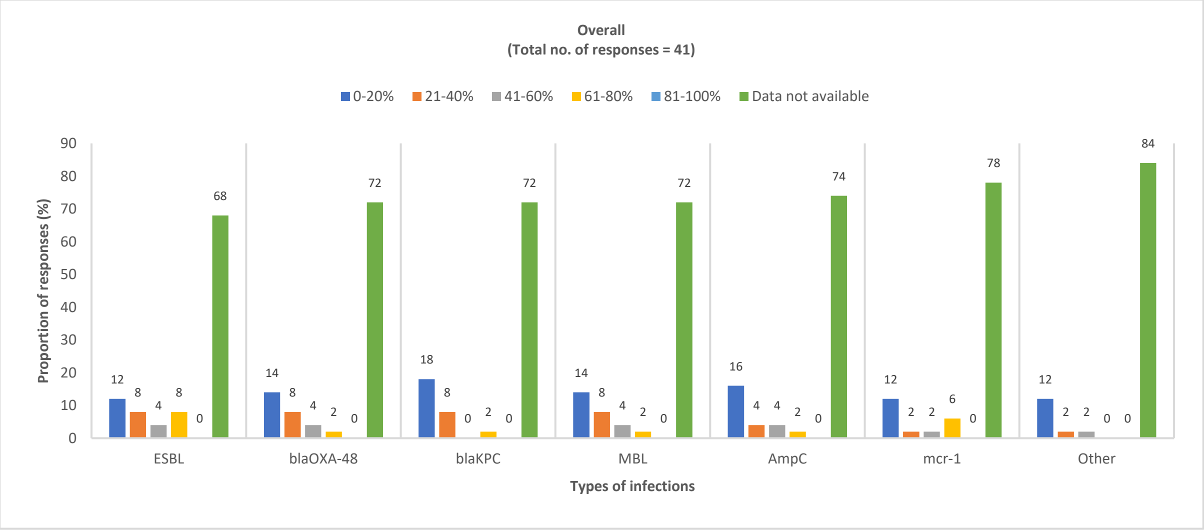

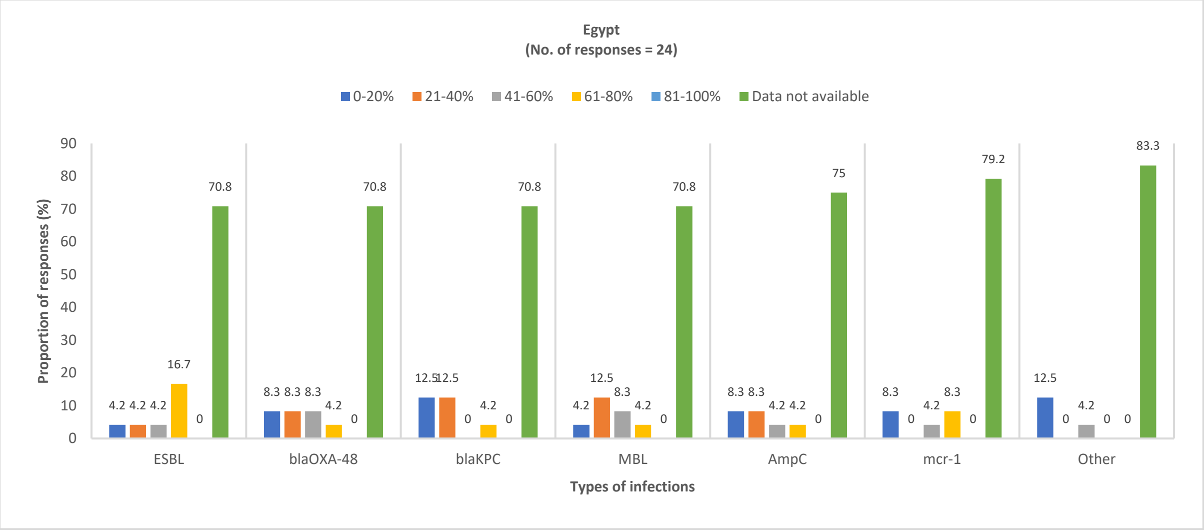

Egypt
(No. of responses = 24)
0-20%
21-40%
41-60%
61-80%
81-100%
Data not available
Proportion of responses (%)
ESBL
blaOXA-48
blaKPC
MBL
AmpC
mcr-1
Other
Types of infections

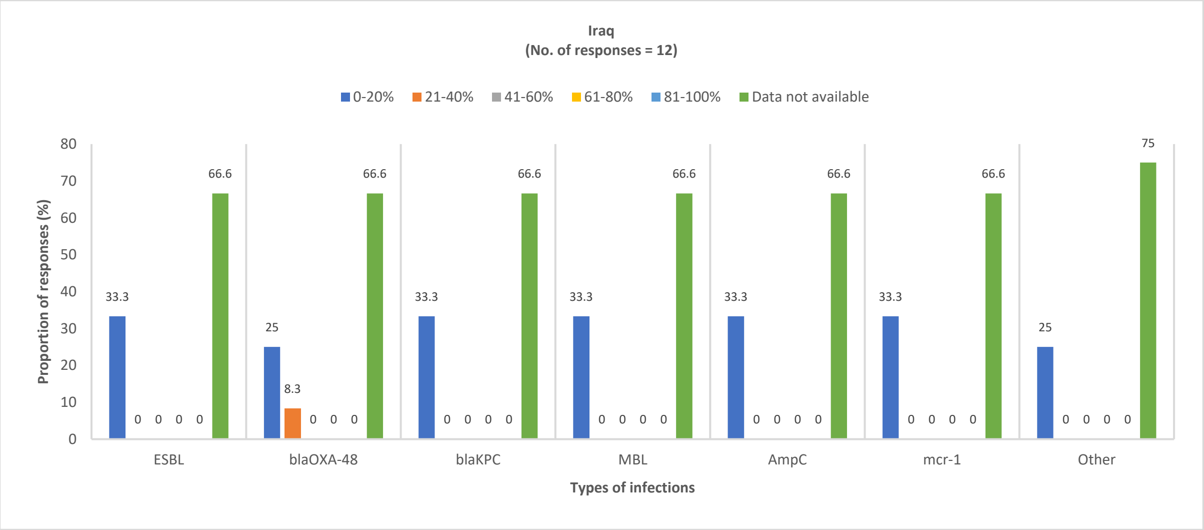

Iraq
(No. of responses = 12)
0-20%
21-40%
41-60%
61-80%
81-100%
Data not available
Proportion of responses (%)
ESBL
blaOXA-48
blaKPC
MBL
AmpC
mcr-1
Other
Types of infections

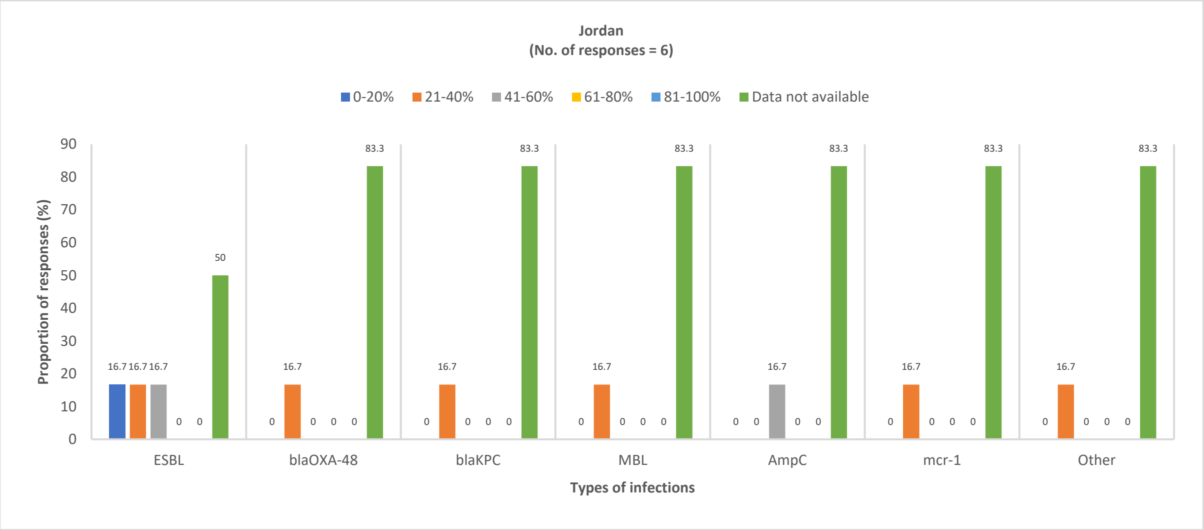

Jordan
(No. of responses = 6)
0-20%
21-40%
41-60%
61-80%
81-100%
Data not available
Proportion of responses (%)
ESBL
blaOXA-48
blaKPC
MBL
AmpC
mcr-1
Other
Types of infections

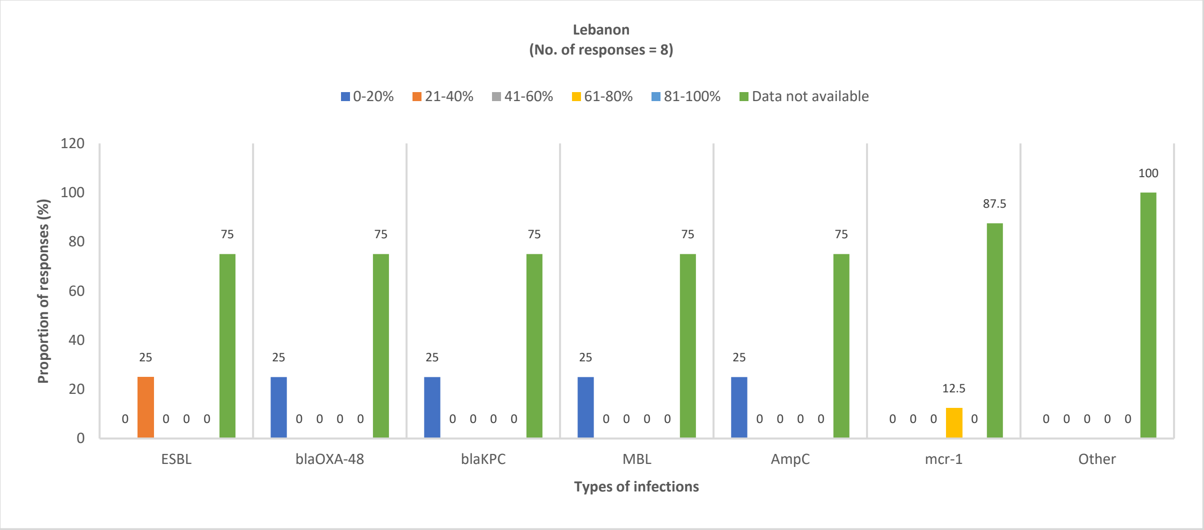
Lebanon
(No. of responses = 8)
0-20%
21-40%
41-60%
61-80%
81-100%
Data not available
Proportion of responses (%)
120
100
80
60
40
20
0
0 25 0 0 0 75
25 0 0 0 0 75
25 0 0 0 0 75
25 0 0 0 0 75
25 0 0 0 0 75
0 0 0 12.5 0 87.5
0 0 0 0 0 100
ESBL
blaOXA-48
blaKPC
MBL
AmpC
mcr-1
Other
Types of infections

Adult

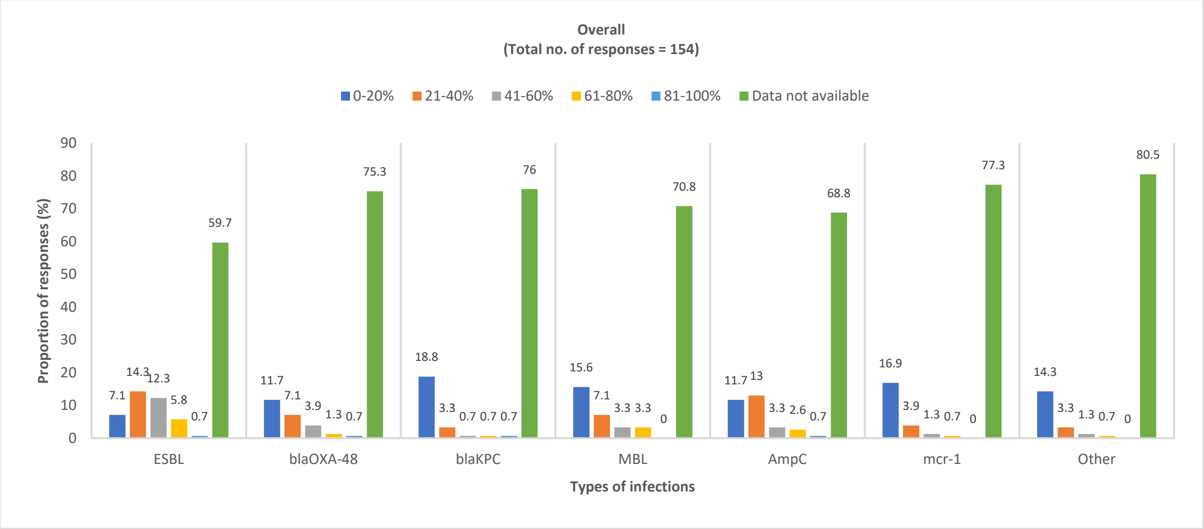
Overall
(Total no. of responses = 154)
0-20%
21-40%
41-60%
61-80%
81-100%
Data not available
Proportion of responses (%)
90
80
70
60
50
40
30
20
10
0
7.1 14.3 12.3 5.8 0.7 59.7
11.7 7.1 3.9 1.3 0.7 75.3
18.8 3.3 0.7 0.7 0.7 76
15.6 7.1 3.3 3.3 0 70.8
11.7 13 3.3 2.6 0.7 68.8
16.9 3.9 1.3 0.7 0 77.3
14.3 3.3 1.3 0.7 0 80.5
ESBL
blaOXA-48
blaKPC
MBL
AmpC
mcr-1
Other
Types of infections

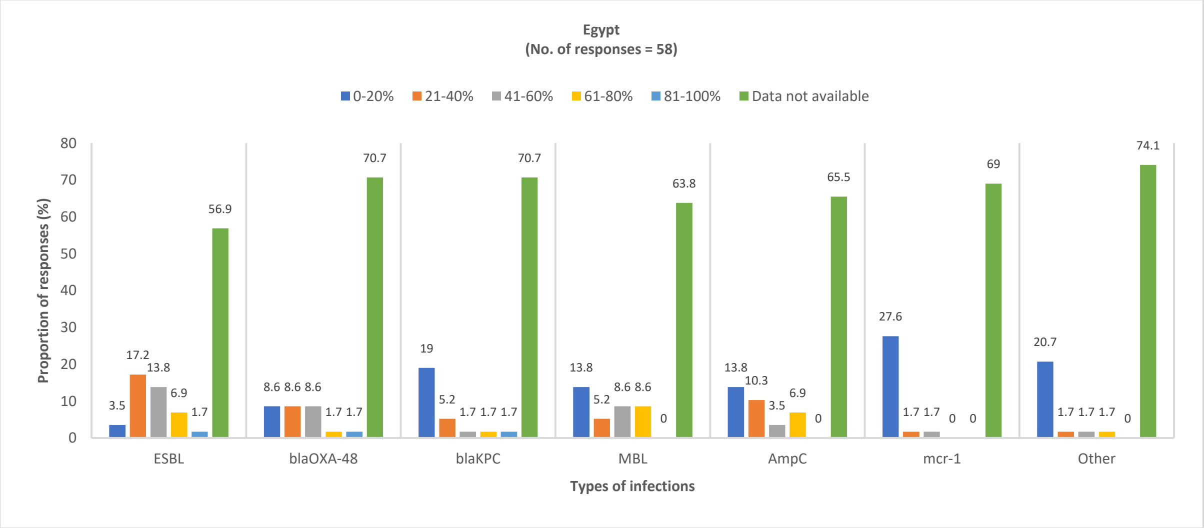
Egypt
(No. of responses = 58)
0-20%
21-40%
41-60%
61-80%
81-100%
Data not available
Proportion of responses (%)
80
70
60
50
40
30
20
10
0
3.5 17.2 13.8 6.9 1.7 56.9
8.6 8.6 8.6 1.7 1.7 70.7
19 5.2 1.7 1.7 1.7 70.7
13.8 5.2 8.6 8.6 0 63.8
13.8 10.3 3.5 6.9 0 65.5
27.6 1.7 1.7 0 0 69
20.7 1.7 1.7 1.7 0 74.1
ESBL
blaOXA-48
blaKPC
MBL
AmpC
mcr-1
Other
Types of infections

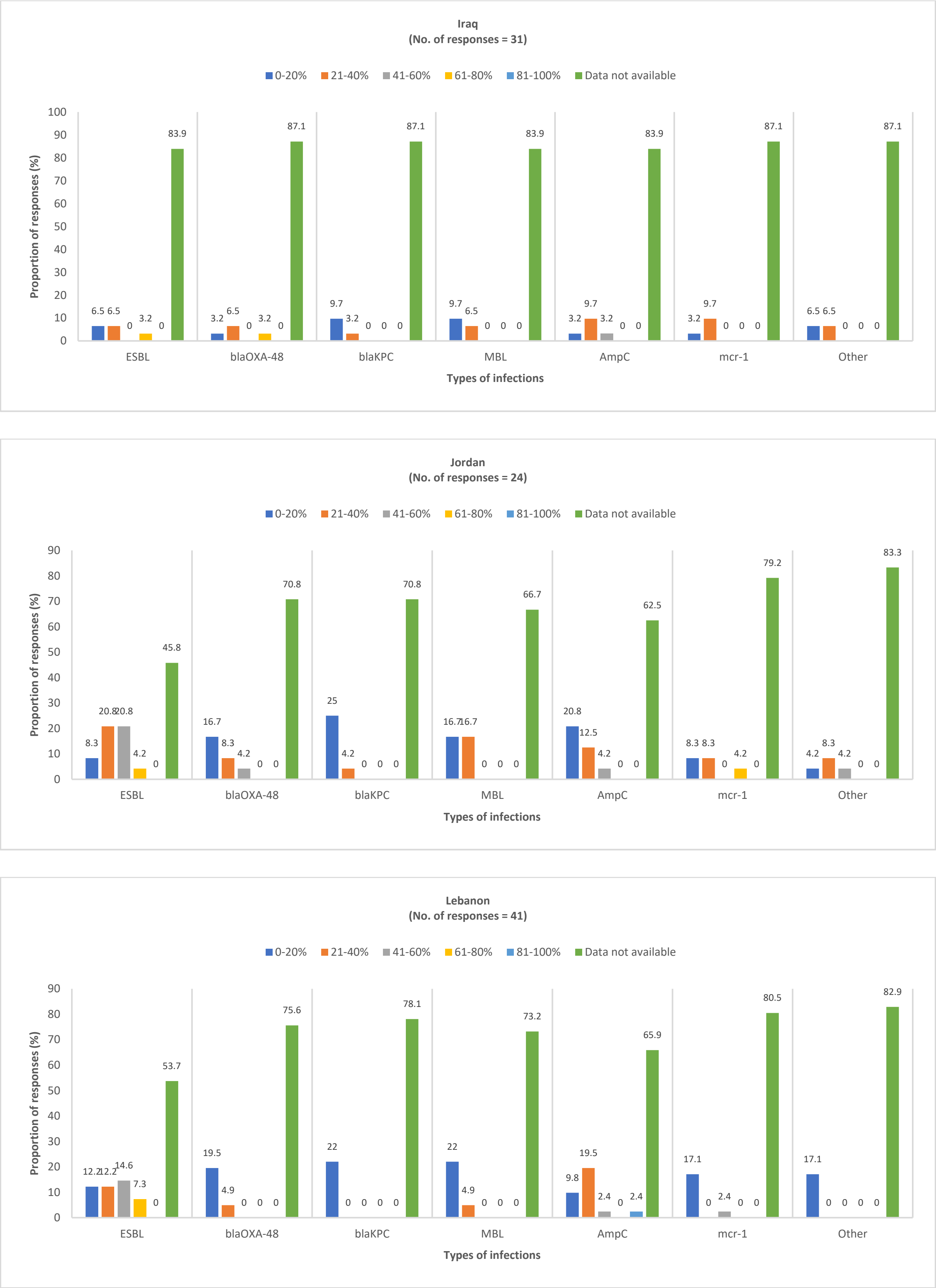

AMR, antimicrobial resistance.

Supplementary Fig 3. Antibiotic use among COVID-19 patients before meeting the physician.

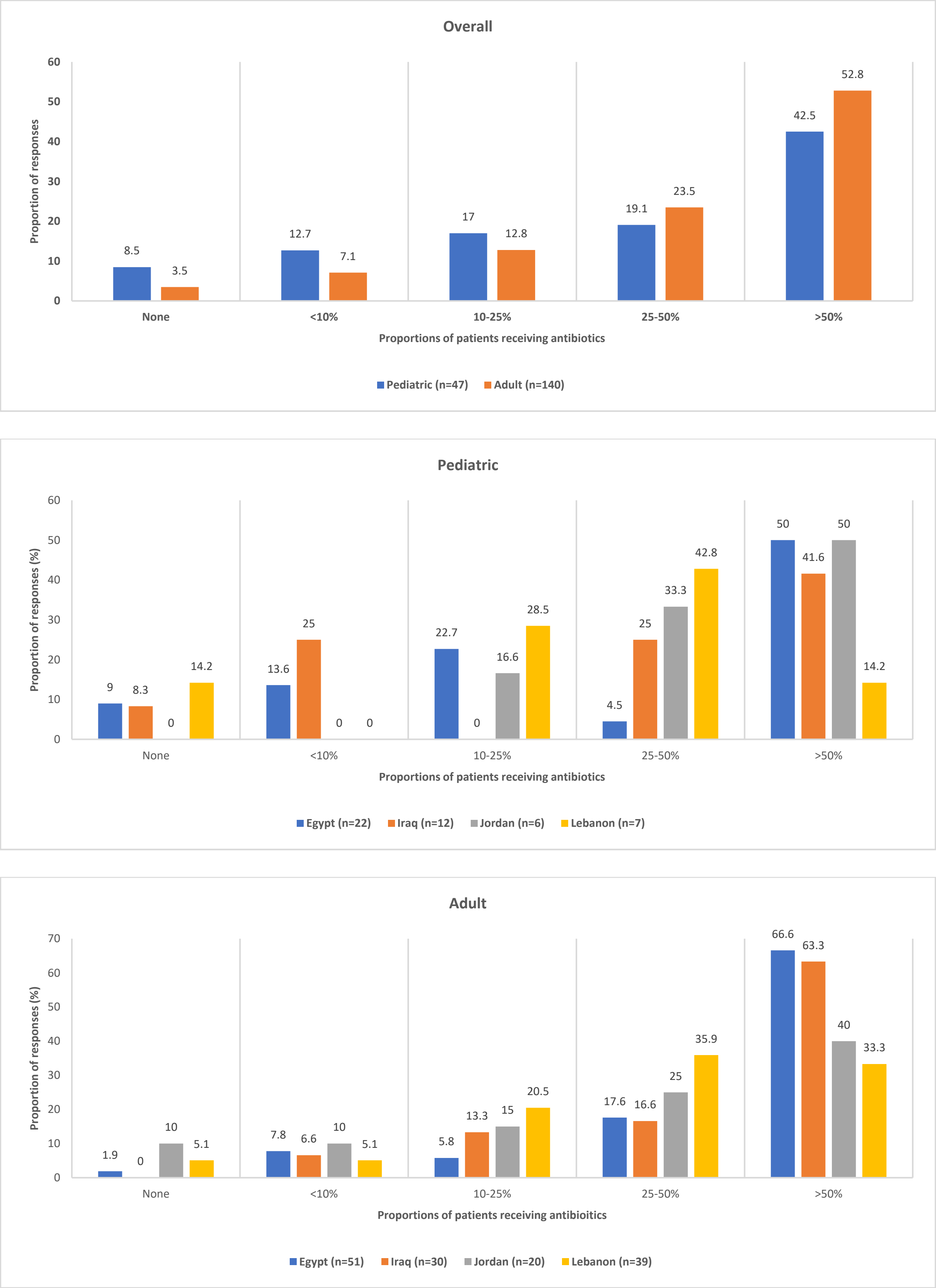

Supplementary Fig 4. Antibiotic use among COVID-19 patients for presumed superimposed bacterial infections.

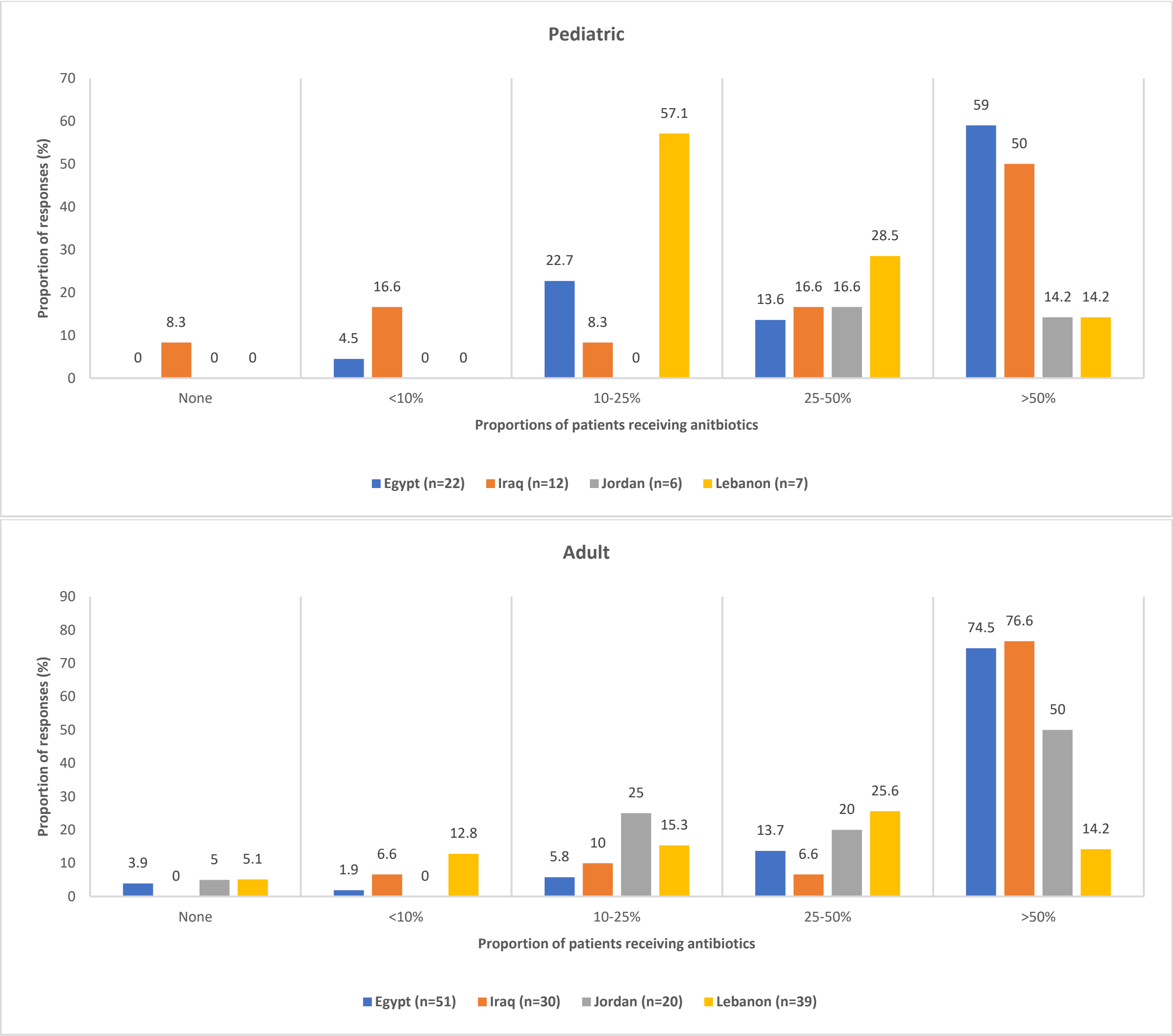

Supplementary Fig 5. Physician perception of the prevalence of multidrug-resistant gram-negative bacteria in COVID intensive-care unit patients.

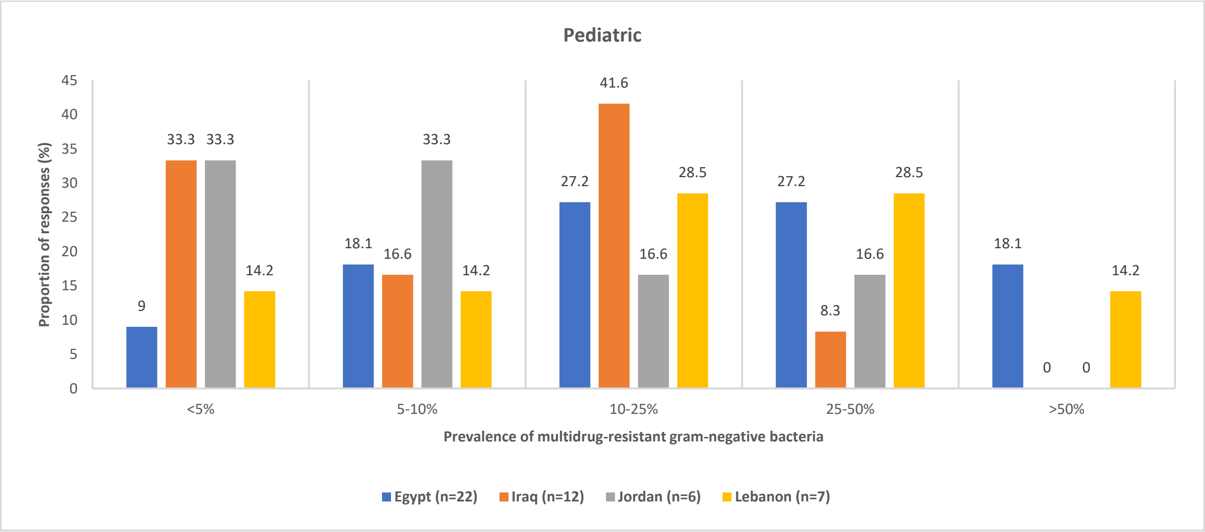

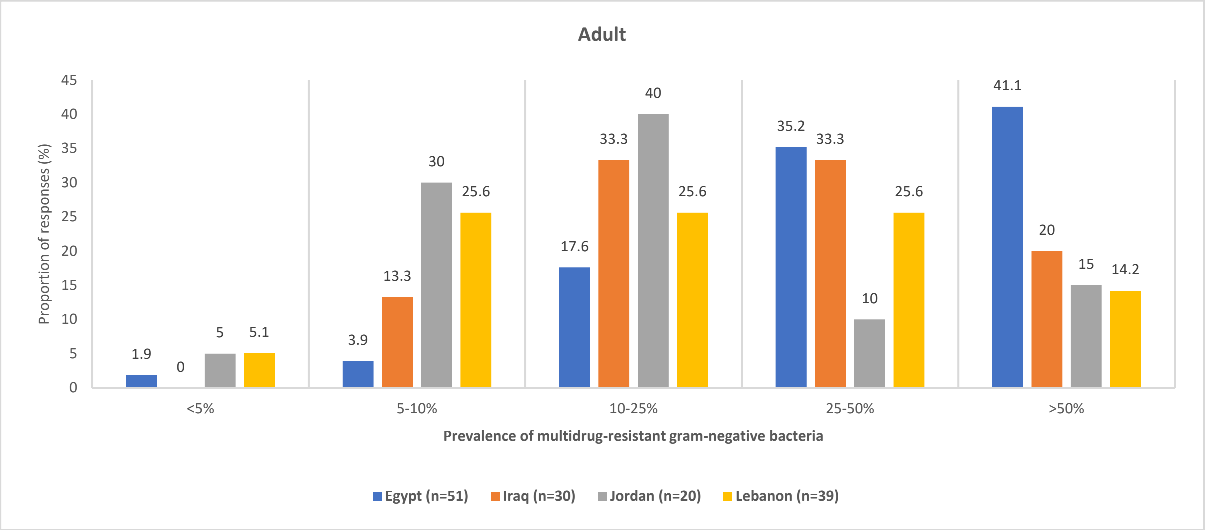

Supplementary Fig 6. Implemented AMS initiatives.

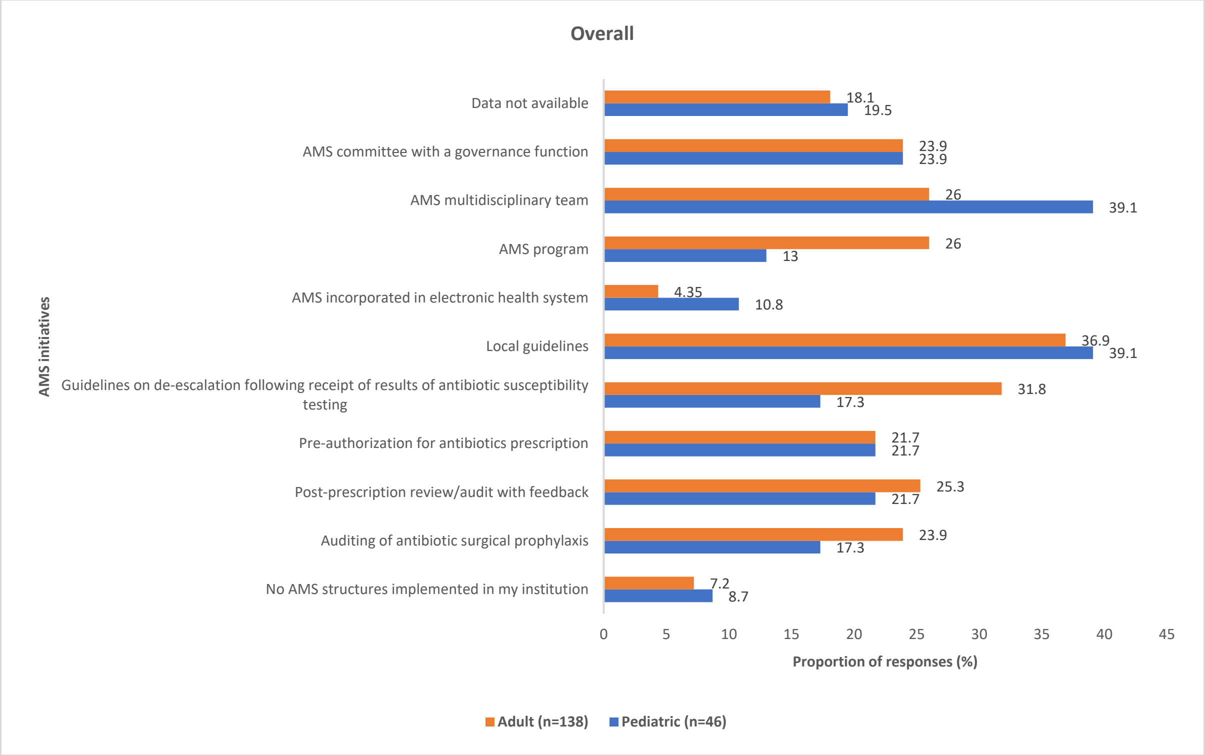

AMS, antimicrobial stewardship.
