# Supplementary material for: Antimicrobial resistance landscape and COVID-19 impact in Egypt, Iraq, Jordan, and Lebanon: A survey-based study and expert opinion
Source: PLoS One. 2023 Jul 27;18(7):e0288550. doi: 10.1371/journal.pone.0288550 (PMC10374138; doi:10.1371/journal.pone.0288550)
Supplement: S2 Appendix — (PDF) [file pone.0288550.s002.pdf]

S2 Appendix - Survey Questionnaire

Antimicrobial Resistance Landscape and COVID-19 Impact E-survey

Dear Doctors,

You have been invited to participate in this web-based online survey on the current antimicrobial resistance (AMR) landscape. The objectives of this survey are to:

- **Understand the current landscape of AMR epidemiology and multidrug resistant (MDR) Gram-negative infection management in adult and pediatric patients**
- **Review available tools and current barriers to effective MDR organism management**
- **Assess the impact of COVID19 on the current AMR landscape and the healthcare systems**
- **Prioritize key action steps for optimizing antimicrobial stewardship (AMS) approaches**

This is a research project being conducted by Pfizer. Your participation in this survey is voluntary and all results are collected and reported anonymously. If you are unable to answer a question, please select 'Data not available' or move on to the next question. By participating in this survey, it is assumed that you agree to the analysis and publication of the survey results. The approximate time needed to complete this survey is 20 - 25 minutes.

Thank you in advance for your time in completing this survey.

Antimicrobial Resistance Landscape and COVID-19 Impact E-survey

Country

* Which country do you work in?

- ◯ Egypt
- ◯ Iraq
- ◯ Jordan
- ◯ Lebanon

Antimicrobial Resistance Landscape and COVID-19 Impact E-survey

Egypt

* Which hospital or medical center in Egypt do you work in?

[ ]

Antimicrobial Resistance Landscape and COVID-19 Impact E-survey

Iraq

* Which hospital or medical center in Iraq do you work in?

Antimicrobial Resistance Landscape and COVID-19 Impact E-survey

Jordan

* Which hospital or medical center in Jordan do you work in?

Antimicrobial Resistance Landscape and COVID-19 Impact E-survey

Lebanon

* Which hospital or medical center in Lebanon do you work in?

Antimicrobial Resistance Landscape and COVID-19 Impact E-survey

Ped vs Adult

* What is your specialty

- ○ Microbiologist
- ○ Intensivist
- ○ Pediatric intensivist
- ○ Neonatologist
- ○ Pediatrician
- ○ Infectious disease specialist
- ○ Pediatric infectious disease specialist
- ○ Chest physician/pulmonologist
- ○ Other

* Which population of patients do you specialize in?

- ○ Pediatric
- ○ Adult

Antimicrobial Resistance Landscape and COVID-19 Impact E-survey

PEDIATRIC - Part 1

Part 1 - Epidemiology and Antimicrobial Resistance

In your hospital, which are the most common risk factors for MDR gram-negative infections in pediatric patients? *(tick all that apply ranking them from 1 [most common risk factor] to 6 [least common risk factor]; **If data is not available, please skip the question.**)*

- Prior infection with MDR gram-negative bacteria
- Colonization with MDR gram-negative bacteria
- Long stay at a healthcare facility (e.g. hospital, nursing home or long-term acute care facility)
- Prior treatment with antibiotics within the last 90 days (3 months)
- Travel or residence in areas with high rates of MDR organisms
- Other

In your hospital, which are the most common risk factors for carbapenem resistant gram-negative infections in pediatric patients? *(tick all that apply ranking them from 1 [most common risk factor] to 11 [least common risk factor]; **If data is not available, please skip the question.**)*

- Previous antibiotic use
- Previous carbapenem use
- Previous colonization
- Mechanical ventilation
- Previous intensive care unit stay
- Dialysis
- Central lines and catheters
- Length of stay in hospital
- ≥2 Comorbidities
- APACHE SCORE II
- Other

In your hospital, MDR bacteria are most frequently encountered in which of the following entities? *(choose all that apply ranking them from 1 [most commonly encountered] to 6 [least commonly encountered]; **If data is not available, please skip the question**.)*

- Lower respiratory tract infections (hospital-acquired pneumonia [HAP]/ventilator-associated pneumonia [VAP])
- Skin and soft tissue infections
- Complicated urinary tract infections (cUTI)
- Catheter-related bloodstream infections (CRBSI)
- Complicated intraabdominal infections (cIAI)
- Others

* In your hospital, what is the prevalence of MDR gram negative bacteria in the following settings (as a % of each infection type)? *(choose one option for each infection type)*

	0-10%	11-20%	21-50%	51-75%	>75%	Data not available
Lower respiratory tract infection (HAP/VAP)	○	○	○	○	○	○
Skin and soft tissue infection	○	○	○	○	○	○
Complicated urinary tract infection	○	○	○	○	○	○
Catheter-related bloodstream infection	○	○	○	○	○	○
Complicated intraabdominal infection	○	○	○	○	○	○

Of all the hospital-acquired infections in your pediatric patients, which of the following Gram negative bacteria are most commonly identified? *(rank the below options from 1 [most commonly identified] to 5 [least commonly identified]; **If data is not available, please skip the question.**)*

- *Acinetobacter baumannii*
- *Escherichia coli*
- *Klebsiella pneumoniae*
- *Pseudomonas aeruginosa*
- Other Enterobacteriaceae

Of these hospital-acquired Gram-negative infections, what is the prevalence of the following resistance mechanisms? *(Provide prevalence of between 0% and 100% using the sliding scale. Please provide accurate data only, if available. Do not include estimates.* ***If the data is not available, please skip the question.****)*

A. baumannii

Extended spectrum β-lactamase (ESBL)-producing A. baumannii

0% 100%

Carbapenem-resistant A. baumannii

0% 100%

E. coli

ESBL-producing E. coli

0% 100%

Carbapenem-resistant E. coli

0% 100%

K. pneumoniae

ESBL-producing K. pneumoniae

0% 100%

Carbapenem-resistant K. pneumoniae

0% 100%

P. aeruginosa

0%	Carbapenem-resistant P. aeruginosa	100%

0%	MDR P. aeruginosa	100%

Other Enterobacteriaceae e.g. *Enterobacter cloacae, Citrobacter species*

0%	Other ESBL-producing Enterobacteriaceae	100%

0%	Other carbapenem-resistant Enterobacteriaceae	100%

* In your hospital, which are the most frequently identified enzyme coding gene mutations leading to antimicrobial resistance in MDR gram-negative species in pediatric patients? *(choose up to 3 options)*

- ☐ ESBL (TEM, SHV, CTX-M)
- ☐ Carbapenemase blaOXA-48
- ☐ Carbapenemase blaKPC
- ☐ Carbapenemase bla MBLs (NDM and VIM)
- ☐ AmpC beta-lactamases
- ☐ *mcr-1* (colistin resistance)
- ☐ Others
- ☐ This information is not available

* In your hospital, what is the prevalence of enzyme coding gene mutations leading to AMR in MDR gram-negative species in pediatric patients?

	0-20%	21-40%	41-60%	61-80%	81-100%	Data not available
ESBL	○	○	○	○	○	○
blaOXA-48	○	○	○	○	○	○
blaKPC	○	○	○	○	○	○
MBL	○	○	○	○	○	○
AmpC	○	○	○	○	○	○
mcr-1	○	○	○	○	○	○
Other	○	○	○	○	○	○

PEDIATRIC - Part 2

Part 2 - MDR Gram-Negative Infections Management

* Does the laboratory in your hospital conduct both genotypic and phenotypic testing of target microorganisms? *(tick one option)*

- ◯ Yes
- ◯ No
- ◯ I don't know

Which of the following are barriers to the successful treatment or medical management of MDR Gram-negative infections in your pediatric population? *(tick all that apply ranking them from 1 [most important barrier] to 7 [least important barrier];* ***If data is not available, please skip the question.****)*

- Limited microbiological diagnostic capabilities
- Availability and cost of newer antibiotics
- The policies and practices inside the hospital
- Lack of knowledge of hospital or national epidemiology and lack of national guidelines
- Administrative challenges
- Lack of an infectious diseases specialist
- Other

What are the barriers to ideal susceptibility testing at your institution/hospital? *(tick all that apply ranking them from 1 [most important barrier] to 8 [least important barrier] ;* ***If data is not available, please skip the question****.)*

- Antimicrobial sensitivity testing technology limitations
- Lack of micro lab capacity (this includes trained microbiologists and resources)
- Lack of adequate sampling
- Lack of timely reporting
- Absence of robust communication policies
- Costs of tests
- Other
- There are no barriers

Which factors do you take into consideration when choosing the appropriate empirical antibiotics therapy? *(tick all that apply ranking them from 1 [most important factor] to 9 [least important factor]; **If data is not available, please skip the question**.)*

- Antibiotic spectrum
- Site of infection
- Comorbidities
- Antibiotic cost
- Antibiotic safety
- Pharmacokinetics/ pharmacodynamics
- Drug-drug interaction
- History of previous antibiotic exposure within last 3 months
- Published guidelines and local antibiograms

* What would be your preferred treatment option for severe infections caused by ESBL-producing Enterobacteriaceae (*E. coli, K. pneumoniae, Enterobacter*, etc.) in your pediatric population? *(select one)*

First Line

- New β-lactam/β-lactamase inhibitor (BL-BLI): ceftazidime-avibactam and ceftolozane-tazobactam
- Conventional BL-BLI e.g. piperacillin-tazobactam, amoxicillin-clavulanic acid, ampicillin-sulbactam
- Aminoglycoside
- Carbapenem
- Polymyxin
- Fluroquinolone
- Monobactam
- Other

* What would be your preferred treatment option for severe infections caused by ESBL-producing Enterobacteriaceae (*E. coli, K. pneumoniae, Enterobacter*, etc.) in your pediatric population? *(select one)*

Second Line

- New β-lactam/β-lactamase inhibitor (BL-BLI): ceftazidime-avibactam and ceftolozane-tazobactam
- Conventional BL-BLI e.g. piperacillin-tazobactam, amoxicillin-clavulanic acid, ampicillin-sulbactam
- Aminoglycoside
- Carbapenem
- Polymyxin
- Fluroquinolone
- Monobactam
- Combination therapy: with aminoglycoside
- Combination therapy: other
- Other

* What would be your preferred treatment option for severe infections caused by carbapenem-resistant Enterobacteriaceae in your pediatric population? *(select one)*

First Line

- ◯ New β-lactam/β-lactamase inhibitor (BL-BLI): ceftazidime-avibactam and ceftolozane-tazobactam
- ◯ Aminoglycoside
- ◯ Polymyxin
- ◯ Fosfomycin
- ◯ Combination therapy: with aminoglycoside
- ◯ Combination therapy: with polymixin
- ◯ Combination therapy: with other agents
- ◯ Other

* What would be your preferred treatment option for severe infections caused by carbapenem-resistant Enterobacteriaceae in your pediatric population? *(select one)*

Second Line

- ◯ New β-lactam/β-lactamase inhibitor (BL-BLI): ceftazidime-avibactam and ceftolozane-tazobactam
- ◯ Aminoglycoside
- ◯ Polymyxin
- ◯ Fosfomycin
- ◯ Combination therapy: with aminoglycoside
- ◯ Combination therapy: with polymixin
- ◯ Combination therapy: with other agents
- ◯ Other

* What would be your preferred treatment option for severe infections caused by carbapenem-resistant Enterobacteriaceae in your pediatric population? *(select one)*

Third Line

- ◯ New β-lactam/β-lactamase inhibitor (BL-BLI): ceftazidime-avibactam and ceftolozane-tazobactam
- ◯ Aminoglycoside
- ◯ Polymyxin
- ◯ Fosfomycin
- ◯ Combination therapy: with aminoglycoside
- ◯ Combination therapy: with polymixin
- ◯ Combination therapy: with other agents
- ◯ Other

* What would be your preferred treatment option for severe infections caused by multidrug resistant *P. aeruginosa* in your pediatric population? *(select one)*

First Line

- ◯ Polymixin
- ◯ Carbapenem
- ◯ Other classical anti-pseudomonal β-lactam (aztreonam, cefipime, ceftazidime or piperacillin-tazobactam)
- ◯ Ceftazidime-avibactam
- ◯ Ceftolozane-tazobactam
- ◯ Combination therapy: colistin with carbapenem/aminoglycoside
- ◯ Combination therapy: aminoglycoside with carbapenem
- ◯ Combination therapy: fosfomycin with carbapenem/β-lactam/aminoglycoside/colistin
- ◯ Other

* What would be your preferred treatment option for severe infections caused by multidrug resistant *P. aeruginosa* in your pediatric population? *(select one)*

Second Line

- Polymixin
- Carbapenem
- Other classical anti-pseudomonal β-lactam (aztreonam, cefipime, ceftazidime or piperacillin-tazobactam)
- Ceftazidime-avibactam
- Ceftolozane-tazobactam
- Combination therapy: colistin with carbapenem/aminoglycoside
- Combination therapy: aminoglycoside with carbapenem
- Combination therapy: fosfomycin with carbapenem/β-lactam/aminoglycoside/colistin
- Other

* What would be your preferred treatment option for severe infections caused by multidrug resistant *P. aeruginosa* in your pediatric population? *(select one)*

Third Line

- Polymixin
- Carbapenem
- Other classical anti-pseudomonal β-lactam (aztreonam, cefipime, ceftazidime or piperacillin-tazobactam)
- Ceftazidime-avibactam
- Ceftolozane-tazobactam
- Combination therapy: colistin with carbapenem/aminoglycoside
- Combination therapy: aminoglycoside with carbapenem
- Combination therapy: fosfomycin with carbapenem/β-lactam/aminoglycoside/colistin
- Other

Antimicrobial Resistance Landscape and COVID-19 Impact E-survey

PEDIATRIC - Part 3

Part 3 – COVID-19 Impact on the AMR Landscape

* In your opinion, has the COVID-19 pandemic impacted AMR and the treatment of gram-negative MDR infections? *(tick one option)*

- Yes
- No
- I don’t know

* In your opinion, has COVID impacted the bacterial epidemiology of your hospital? *(tick one option)*

- Yes
- Yes, only the ICU
- No
- I don’t know

* From your experience in your hospital, has a higher incidence of AMR been observed in patients with active or past COVID-19 infection? (tick one option)

- ◯ Yes
- ◯ No
- ◯ Data not available

* Which factors have impacted the treatment of gram-negative MDR infections in your hospital during the COVID-19 pandemic? *(tick all that apply)*

- ☐ Interrupted supply chains, drug shortages or stock outs
- ☐ Changes in AMR epidemiology
- ☐ Deprioritisation of AMR surveillance and AMS
- ☐ Full hospital occupancy
- ☐ All of the above
- ☐ I don’t know
- ☐ None of the above

* How many of your COVID-19 patients were on antibiotics before you attended to their care? *(tick one option)*

- ◯ None
- ◯ <10%
- ◯ 10-25%
- ◯ 25-50%
- ◯ >50%

* In your practice, how many COVID patients received antibiotics for presumed super imposed bacterial infection? *(tick one option)*

- ◯ None
- ◯ <10%
- ◯ 10-25%
- ◯ 25-50%
- ◯ >50%

* In your practice, how frequently have you encountered MDR gram-negative bacteria in COVID ICU patients? *(tick one option)*

- ◯ <5%
- ◯ 5-10%
- ◯ 10-25%
- ◯ 25-50%
- ◯ >50%

Antimicrobial Resistance Landscape and COVID-19 Impact E-survey

PEDIATRIC - Part 4

Part 4 - Antimicrobial Stewardship (AMS) Implementation

* Which of the following AMS structures are implemented in your institution? *(tick all options that apply)*

- [ ] AMS committee with a governance function
- [ ] AMS multidisciplinary team
- [ ] AMS program
- [ ] AMS incorporated in electronic health system
- [ ] Local guidelines
- [ ] Guidelines on de-escalation following receipt of results of antibiotic susceptibility testing
- [ ] Pre-authorization for antibiotics prescription
- [ ] Post-prescription review/audit with feedback
- [ ] Auditing of antibiotic surgical prophylaxis
- [ ] No AMS structures implemented in my institution
- [ ] Data not available

What are the main barriers to AMS implementation in your hospital? *(tick all that apply ranking them from 1 [most important barrier] to 10 [least important barrier]; **If data is not available, please skip the question.**)*

- Lack of adequate staff for AMS
- Lack of education and training of healthcare facility staff on AMS
- Lack of laboratory resources to culture and test isolates for drug susceptibility
- Absence of adequate funding for development of infrastructure
- Lack of regional and national surveillance systems
- Lack of automated system to track antibiotic prescriptions and AMR surveillance
- Miscommunication between different specialties
- Poor understanding of AMS among HCPs
- Lack of commitment of higher administration
- None of the above

* When do you order culture and antibiotic susceptibility testing of bacterial pathogens within your hospital/center? *(tick one option)*

- ○ Test all infected ICU patients
- ○ Test only patients at high risk of MDR infection
- ○ Test only patients failing initial therapy
- ○ Test all patients requiring antibiotic treatment
- ○ No antibiotic susceptibility testing is available
- ○ Other

Antimicrobial Resistance Landscape and COVID-19 Impact E-survey

ADULT - Part 1

Part 1 - Epidemiology and Antimicrobial Resistance

In your hospital, which are the most common risk factors for MDR gram-negative infections in adult patients? (tick all that apply ranking them from 1 [most common risk factor] to 7 [least common risk factor]; **If data is not available, please skip the question.**)

- Prior infection with MDR gram-negative bacteria
- Colonization with MDR gram-negative bacteria
- Long stay at a healthcare facility (e.g. hospital, nursing home or long-term acute care facility)
- Prior treatment with antibiotics within the last 90 days (3 months)
- Travel or residence in areas with high rates of MDR organisms
- The patient is a health-care professional
- Other

In your hospital, which are the most common risk factors for carbapenem resistant gram-negative infections in adult patients? *(tick all that apply ranking them from 1 [most common risk factor] to 11 [least common risk factor]; **If data is not available, please skip the question.**)*

- Previous antibiotic use
- Previous carbapenem use
- Previous colonization
- Mechanical ventilation
- Previous intensive care unit stay
- Dialysis
- Central lines and catheters
- Length of stay in hospital
- ≥2 Comorbidities
- APACHE SCORE II
- Other

In your hospital, MDR bacteria are most frequently encountered in which of the following entities? *(choose all that apply ranking them from 1 [most commonly encountered] to 6 [least commonly encountered]; **If data is not available, please skip the question.**)*

- Lower respiratory tract infection (hospital-acquired pneumonia [HAP]/ventilator-associated pneumonia [VAP])
- Skin and soft tissue infection
- Complicated urinary tract infection (cUTI)
- Catheter-related bloodstream infection (CRBSI)
- Complicated intraabdominal infection (cIAI)

* In your hospital, what is the prevalence of MDR gram negative bacteria in the following settings (as a % of each infection type)? *(choose one option for each infection type)*

	0-10%	11-20%	21-50%	51-75%	>75%	Data not available
Lower respiratory tract infection (HAP/VAP)	○	○	○	○	○	○
Skin and soft tissue infection	○	○	○	○	○	○
Complicated urinary tract infection	○	○	○	○	○	○
Catheter-related bloodstream infection	○	○	○	○	○	○
Complicated intraabdominal infection	○	○	○	○	○	○

Of all the hospital-acquired infections in your adult patients, which of the following Gram negative bacteria are most commonly identified? *(rank the below options from 1 [most commonly identified] to 5 [least commonly identified]; **If data is not available, please skip the question.**)*

- *Acinetobacter baumannii*
- *Escherichia coli*
- *Klebsiella pneumoniae*
- *Pseudomonas aeruginosa*
- Other Enterobacteriaceae

Of these hospital-acquired Gram-negative infections, what is the prevalence of the following resistance mechanisms in your adult patients? *(Provide prevalence of between 0% and 100% using the sliding scale. Please provide accurate data only, if available. Do not include estimates.* ***If the data is not available, please skip the question.****)*

A. baumannii

Extended spectrum β-lactamase (ESBL)-producing A. baumannii

0% 100%

Carbapenem-resistant A. baumannii

0% 100%

E. coli

ESBL-producing E. coli

0% 100%

Carbapenem-resistant E. coli

0% 100%

K. pneumoniae

ESBL-producing K. pneumoniae

0% 100%

Carbapenem-resistant K. pneumoniae

0% 100%

P. aeruginosa

Carbapenem-resistant P. aeruginosa

0% — 100% [ ]

MDR P. aeruginosa

0% — 100% [ ]

Other Enterobacteriaceae e.g. *Enterobacter cloacae, Citrobacter species*

Other ESBL-producing Enterobacteriaceae

0% — 100% [ ]

Other carbapenem-resistant Enterobacteriaceae

0% — 100% [ ]

* In your hospital, which are the most frequently identified enzyme coding gene mutations leading to AMR in MDR gram-negative species in adult patients? *(choose top 3 options)*

- [ ] ESBL (TEM, SHV, CTX-M)
- [ ] Carbapenemase *bla*OXA-48
- [ ] Carbapenemase *bla*KPC
- [ ] Carbapenemase bla MBLs (NDM and VIM)
- [ ] AmpC beta-lactamases
- [ ] *mcr-1* (colistin resistance)
- [ ] Others
- [ ] Data not available

* In your hospital, what is the prevalence of enzyme coding gene mutations leading to AMR in MDR gram-negative species in adult patients?

	0-20%	21-40%	41-60%	61-80%	81-100%	Data not available
ESBL	○	○	○	○	○	○
*bla*OXA-48	○	○	○	○	○	○
*bla*KPC	○	○	○	○	○	○
MBL	○	○	○	○	○	○
AmpC	○	○	○	○	○	○
mcr-1	○	○	○	○	○	○
Other	○	○	○	○	○	○

ADULT - Part 2

Part 2 - MDR Gram-Negative Infections Management

* Does the laboratory in your hospital conduct both genotypic and phenotypic testing of target microorganisms? *(tick one option)*

- Yes
- No
- I don't know

Which of the following are barriers to the successful treatment or medical management of MDR Gram-negative infections in your adult population? *(tick all that apply ranking them from 1 [most important barrier] to 7 [least important barrier]; **If data is not available, please skip the question.)***

- Limited microbiological diagnostic capabilities
- Availability and cost of newer antibiotics
- The policies and practices inside the hospital
- Lack of knowledge of hospital or national epidemiology and lack of national guidelines
- Administrative challenges
- Lack of an infectious diseases specialist
- Other

What are the barriers to ideal susceptibility testing at your institution/hospital? *(tick all that apply ranking them from 1 [most important barrier] to 8 [least important barrier]; **If data is not available, please skip the question.**)*

- Antimicrobial sensitivity testing technology limitations
- Lack of micro lab capacity (this includes trained microbiologists and resources)
- Lack of adequate sampling
- Lack of timely reporting
- Absence of robust communication policies
- Costs of tests
- Other
- There are no barriers

Which factors do you take into consideration when choosing the appropriate empirical antibiotics therapy? *(tick all that apply ranking them from 1 [most important factor] to 9 [least important factor]; **If data is not available, please skip the question.**)*

- Antibiotic spectrum
- Site of infection
- Comorbidities
- Antibiotic cost
- Antibiotic safety
- Pharmacokinetics/ pharmacodynamics
- Drug-drug interaction
- History of previous antibiotic exposure within last 3 months
- Published guidelines and local antibiograms

* What would be your preferred treatment option for severe infections caused by ESBL-producing Enterobacteriaceae (*E. coli, K. pneumoniae, Enterobacter*, etc.) in your adult population? *(select one)*

First Line

- New β-lactam/β-lactamase inhibitor (BL-BLI): ceftazidime-avibactam and ceftolozane-tazobactam
- Conventional BL-BLI e.g. piperacillin-tazobactam, amoxicillin-clavulanic acid, ampicillin-sulbactam
- Aminoglycoside
- Carbapenem
- Polymyxin
- Fluroquinolone
- Monobactam
- Other

* What would be your preferred treatment option for severe infections caused by ESBL-producing Enterobacteriaceae (*E. coli, K. pneumoniae, Enterobacter*, etc.) in your adult population? *(select one)*

Second Line

- New β-lactam/β-lactamase inhibitor (BL-BLI): ceftazidime-avibactam and ceftolozane-tazobactam
- Conventional BL-BLI
- Aminoglycoside
- Carbapenem
- Polymyxin
- Fluroquinolone
- Monobactam
- Combination therapy: with aminoglycoside
- Combination therapy: with aminoglycoside
- Other

* What would be your preferred treatment option for severe infections caused by carbapenem-resistant Enterobacteriaceae in your adult population? *(select one)*

First Line

- New β-lactam/β-lactamase inhibitor (BL-BLI): ceftazidime-avibactam and ceftolozane-tazobactam
- Aminoglycoside
- Polymyxin
- Tigecycline
- Fosfomycin
- Combination therapy: with aminoglycoside
- Combination therapy: with polymyxin
- Combination therapy: with other agents
- Other

* What would be your preferred treatment option for severe infections caused by carbapenem-resistant Enterobacteriaceae in your adult population? *(select one)*

Second Line

- New β-lactam/β-lactamase inhibitor (BL-BLI): ceftazidime-avibactam and ceftolozane-tazobactam
- Aminoglycoside
- Polymyxin
- Tigecycline
- Fosfomycin
- Combination therapy: with aminoglycoside
- Combination therapy: with polymyxin
- Combination therapy: with other agents
- Other

* What would be your preferred treatment option for severe infections caused by carbapenem-resistant Enterobacteriaceae in your adult population? *(select one)*

Third Line

- New β-lactam/β-lactamase inhibitor (BL-BLI): ceftazidime-avibactam and ceftolozane-tazobactam
- Aminoglycoside
- Polymyxin
- Tigecycline
- Fosfomycin
- Combination therapy: with aminoglycoside
- Combination therapy: with polymyxin
- Combination therapy: with other agents
- Other

* What would be your preferred treatment option for severe infections caused by multidrug resistant *P. aeruginosa* in your adult population? *(select one)*

First Line

- Polymixin
- Carbapenem
- Other classical anti-pseudomonal β-lactam (aztreonam, cefipime, ceftazidime or piperacillin-tazobactam)
- Ceftazidime-avibactam
- Ceftolozane-tazobactam
- Combination therapy: colistin with carbapenem/aminoglycoside
- Combination therapy: aminoglycoside with carbapenem
- Combination therapy: fosfomycin with carbapenem/β-lactam/aminoglycoside/colistin
- Other

* What would be your preferred treatment option for severe infections caused by multidrug resistant *P. aeruginosa* in your adult population? *(select one)*

Second Line

- Polymixin
- Carbapenem
- Other classical anti-pseudomonal β-lactam (aztreonam, cefipime, ceftazidime or piperacillin-tazobactam)
- Ceftazidime-avibactam
- Ceftolozane-tazobactam
- Combination therapy: colistin with carbapenem/aminoglycoside
- Combination therapy: aminoglycoside with carbapenem
- Combination therapy: fosfomycin with carbapenem/β-lactam/aminoglycoside/colistin
- Other

* What would be your preferred treatment option for severe infections caused by multidrug resistant *P. aeruginosa* in your adult population? *(select one)*

Third Line

- Polymixin
- Carbapenem
- Other classical anti-pseudomonal β-lactam (aztreonam, cefipime, ceftazidime or piperacillin-tazobactam)
- Ceftazidime-avibactam
- Ceftolozane-tazobactam
- Combination therapy: colistin with carbapenem/aminoglycoside
- Combination therapy: aminoglycoside with carbapenem
- Combination therapy: fosfomycin with carbapenem/β-lactam/aminoglycoside/colistin
- Other

Antimicrobial Resistance Landscape and COVID-19 Impact E-survey

ADULT - Part 3

Part 3 – COVID-19 Impact on the AMR Landscape

* In your opinion, has the COVID-19 pandemic impacted AMR and the treatment of gram-negative MDR infections? *(tick one option)*

- Yes
- No
- I don’t know

* In your opinion, has COVID impacted the bacterial epidemiology of your hospital? *(tick one option)*

- Yes
- Yes, only the ICU
- No
- I don’t know

* From your experience in your hospital, has a higher incidence of AMR been observed in patients with active or past COVID-19 infection? *(tick one option)*

- Yes
- No
- Data not available

* Which factors have impacted the treatment of gram-negative MDR infections in your hospital during the COVID-19 pandemic? *(tick all that apply)*

- Interrupted supply chains, drug shortages or stock outs
- Changes in AMR epidemiology
- Deprioritisation of AMR surveillance and AMS
- Full hospital occupancy
- All of the above
- Data not available
- None of the above

* How many of your COVID-19 patients were on antibiotics before you attended to their care? *(tick one option)*

- None
- <10%
- 10-25%
- 25-50%
- >50%

* In your practice, how many COVID patients received antibiotics for presumed super imposed bacterial infection? *(tick one option)*

- None
- <10%
- 10-25%
- 25-50%
- >50%

* In your practice, how frequently have you encountered MDR gram-negative bacteria in COVID ICU patients? *(tick one option)*

- None
- <10%
- 10-25%
- 25-50%
- >50%

ADULT - Part 4

Part 4 - Antimicrobial Stewardship Implementation

* Which of the following AMS structures are implemented in your institution? *(tick all options that apply)*

- ☐ AMS committee with a governance function
- ☐ AMS multidisciplinary team
- ☐ AMS program
- ☐ AMS incorporated in electronic health system
- ☐ Local guidelines
- ☐ Guidelines on de-escalation following receipt of results of antibiotic susceptibility testing
- ☐ Pre-authorization for antibiotics prescription
- ☐ Post-prescription review/audit with feedback
- ☐ Auditing of antibiotic surgical prophylaxis
- ☐ No AMS structures implemented in my institution
- ☐ Data not available

What are the main barriers to AMS implementation in your hospital? *(tick all that apply ranking them from 1 [most important barrier] to 11 [least important barrier];* ***If data is not available, please skip the question.****)*

- Lack of adequate staff for AMS
- Lack of education and training of healthcare facility staff on AMS
- Lack of laboratory resources to culture and test isolates for drug susceptibility
- Absence of adequate funding for development of infrastructure
- Lack of regional and national surveillance systems
- Lack of automated system to track antibiotic prescriptions and AMR surveillance
- Miscommunication between different specialties
- Poor understanding of AMS among HCPs
- Lack of commitment of higher administration
- None of the above
- Data not available

* When do you generally order culture and antibiotic susceptibility testing of bacterial pathogens within your hospital/center? *(tick one option)*

- ○ Test all infected ICU patients
- ○ Test only patients at high risk of MDR infection
- ○ Test only patients failing initial therapy
- ○ Test all patients requiring antibiotic treatment
- ○ No antibiotic susceptibility testing is available
- ○ Other

Antimicrobial Resistance Landscape and COVID-19 Impact E-survey

Than you Page

Thank you for taking our survey.
